# The oncogenicity of tumor-derived mutant p53 is enhanced by the recruitment of PLK3

**DOI:** 10.1038/s41467-021-20928-8

**Published:** 2021-01-29

**Authors:** Catherine A. Vaughan, Shilpa Singh, Mark A. Subler, Jolene J. Windle, Kazushi Inoue, Elizabeth A. Fry, Raghavendra Pillappa, Steven R. Grossman, Brad Windle, W. Andrew Yeudall, Swati Palit Deb, Sumitra Deb

**Affiliations:** 1 Philips Institute, Virginia Commonwealth University, Richmond, VA 23298 USA; 2 Department of Human and Molecular Genetics, Virginia Commonwealth University, Richmond, VA 23298 USA; 3 Department of Pathology, Wake Forest School of Medicine, Winston-Salem, NC 27101 USA; 4 Department of Pathology, Virginia Commonwealth University, Richmond, VA 23298 USA; 5 Massey Cancer Center, Virginia Commonwealth University, Richmond, VA 23298 USA; 6 Department of Internal Medicine, Division of Hematology, Oncology and Palliative Care, Virginia Commonwealth University, Richmond, VA 23298 USA; 7 Department of Oral Biology & Diagnostic Sciences, Augusta University, Augusta, GA 30912 USA; 8 Georgia Cancer Center, Augusta University, Augusta, GA 30912 USA; 9 Department of Biochemistry & Molecular Biology, Virginia Commonwealth University, Richmond, VA 23298 USA

**Keywords:** Lung cancer, Tumour-suppressor proteins, Mechanisms of disease

## Abstract

p53 mutations with single amino acid changes in cancer often lead to dominant oncogenic changes. Here, we have developed a mouse model of gain-of-function (GOF) p53-driven lung cancer utilizing conditionally active LSL p53-R172H and LSL K-Ras-G12D knock-in alleles that can be activated by Cre in lung club cells. Mutation of the p53 transactivation domain (TAD) (p53-L25Q/W26S/R172H) eliminating significant transactivation activity resulted in loss of tumorigenicity, demonstrating that transactivation mediated by or dependent on TAD is required for oncogenicity by GOF p53. GOF p53 TAD mutations significantly reduce phosphorylation of nearby p53 serine 20 (S20), which is a target for PLK3 phosphorylation. Knocking out PLK3 attenuated S20 phosphorylation along with transactivation and oncogenicity by GOF p53, indicating that GOF p53 exploits PLK3 to trigger its transactivation capability and exert oncogenic functions. Our data show a mechanistic involvement of PLK3 in mutant p53 pathway of oncogenesis.

## Abstract (web summary)

The mechanisms of how gain-of-function (GOF) mutant p53 drives carcinogenesis are unclear. Here, the authors show that a GOF mutant p53 requires its transactivation capability to induce mouse lung tumors and this is dependent on PLK3 phosphorylation of GOF mutant p53.

## Introduction

p53 mutations in cancer often lead to single amino acid changes, with many of these missense mutants possessing gain-of-function (GOF) properties^1^. The GOF p53 mutants not only lose their tumor suppressor activity (loss-of-function), but also gain new oncogenic functions that give cancer cells a growth advantage over their normal counterparts^2–5^. Expression of GOF p53 mutants induces several oncogenic and/or proliferative activities such as increased cell growth, motility, invasion, tumorigenicity, and metastatic ability of cancer cells^6–10^, amongst others. GOF activity of endogenous mutant p53 in cancer cells has been demonstrated by depleting GOF p53 using RNAi^4,11,12^. Several laboratories have also used knock-in mouse models to demonstrate GOF activity^10,13–16^.

Simply, p53 mutations can be divided into three groups^17,18^: (1) loss of function, where p53 mutation leads to loss of tumor suppressor function of p53, (2) dominant negative, where a mutant copy hetero-oligomerizes with a wild-type (WT) copy poisoning the WT tumor suppressor function, and (3) GOF, where the cells acquire a new dominant oncogenic property leading to oncogenesis.

One mechanism of GOF by mutant p53 has been thought to be mutant p53-mediated transactivation of a series of proliferation- and oncogenesis-related genes^19,20^ after activation following phosphorylation. It is, in general, speculated that mutant p53 upregulates gene expression by its nucleation on the target promoter via its interaction with a transcription factor (TF)^17,19,21^. While interaction with individual TFs in cultured cells has often been implicated in transcriptional activation^4,17,19,22^, the communication scheme of GOF p53 with the TFs on target gene promoters needed for transactivation is elusive so far, and until now there is no evidence demonstrating that tumorigenicity induced by GOF p53 is mediated by its transactivation function in vivo.

Human Mediator complex is a large transcriptional complex consisting of Mediator subunits which functions in transcription as coactivators through interactions with TFs and general transcriptional machinery^23^. It is reported that in activated transcription, mediated by WT p53, p53 interacts with Med17 by its N-terminal transactivation domain (TAD) and with Med1 by its C-terminal domain^24^, and WT p53 induces a structural shift within the mediator. Mutations in WT p53 TAD at amino acids 22 and 23 not only prevent transactivation of many genes, but also inhibit the interaction between WT p53 and Med17^24,25^.

PLK3 is a member of a family of serine–threonine kinases that play vital roles in the cell cycle^26^. Under conditions of DNA damage, PLK3 has been shown to interact and phosphorylate WT p53 at S20; it activates the tumor suppressor inducing apoptosis^27,28^. The limited analysis that has been carried out on PLK3 in cancer samples indicates that its expression is somewhat deregulated in a number of cancers^26^.

We have developed a lung tumor mouse model by expressing GOF p53-R172H and K-Ras (G12D) knock-ins in Club (bronchial-epithelial) cells, in which tumor formation can be prevented by doxycycline (Dox)-inducible GOF p53 depletion. A matched K-Ras and transactivation-deficient GOF p53 knock-in mouse model, where GOF p53 harbored three amino acid substitutions at positions L25Q/W26S/R172H, was defective in generating lung tumors. A lesion in TAD caused substantial loss of GOF activity. Since the TAD domain of p53 is activated by phosphorylation^29^, we also examined the effect of the two amino acid substitutions in TAD on phosphorylation of mutant p53. Indeed, TAD-mutant p53 shows a reduction of phosphorylation at S20. Depletion of PLK3 lowers S20 phosphorylation, transactivation, and oncogenicity by mutant p53, suggesting PLK3-induced S20 phosphorylation plays a vital role in mutant p53-mediated oncogenesis.

## Results

### Lung tumor formation in the GOF p53-expressing lung cancer mouse model is dependent on expression of mutant p53

**Fig. 1:**
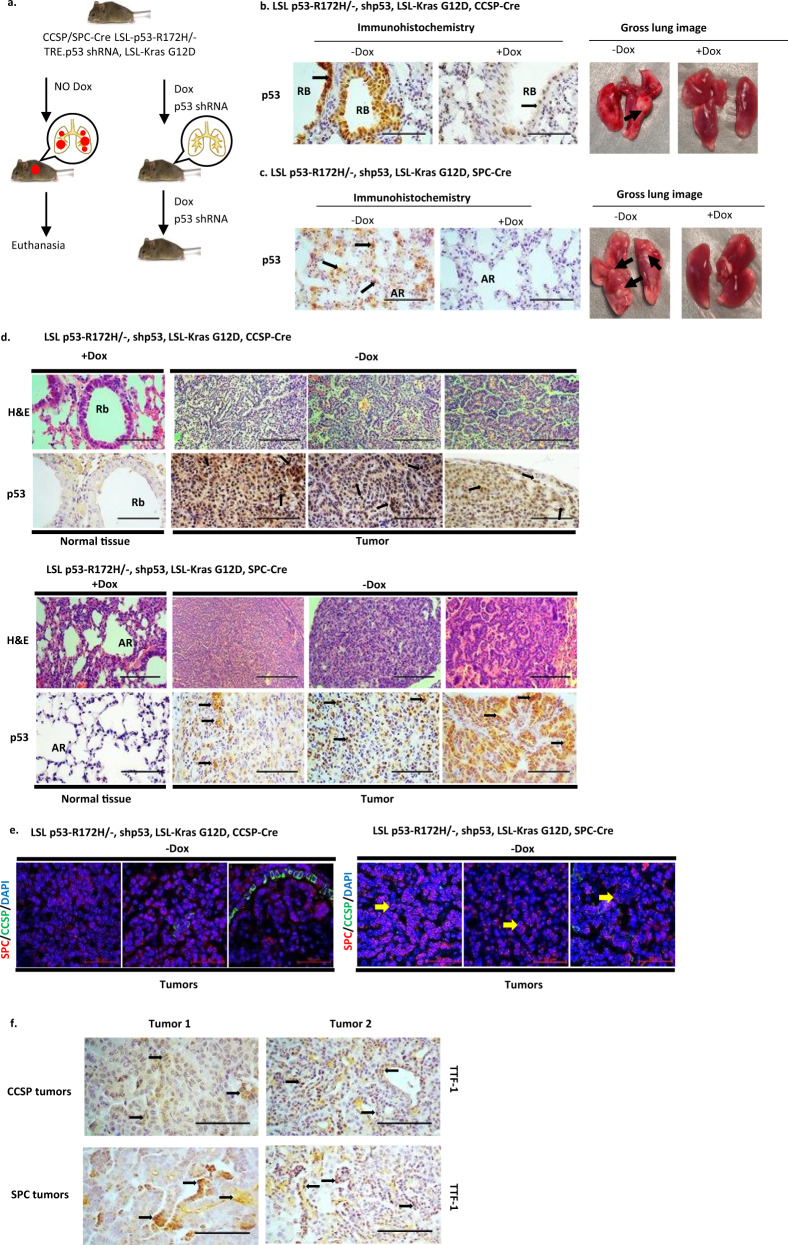
Lung tumor formation in the GOF p53-expressing mouse lung cancer model is dependent on expression of mutant p53. **a** Model shows induced depletion of p53 causes tumor remission. Induction of p53 shRNA causes a reduction of p53-R172H in the mouse lung after dox treatment. Dox was added to the drinking water when mice were 1 month old and continued throughout the life of the mice. **b** Representative images (Magnification: 20×, Scale bar: 100 µM) of tissue sections from the LSL p53-R172H, shp53, K-Ras, CCSP-Cre mouse line showing apparently normal lung Club cells immunostained for p53 (arrows) in small bronchi lined by respiratory epithelium; +Dox shows a decrease in p53 expression indicated by reduced staining intensity; gross lung image shows formation of lung tumor (shown by a black arrow) in –Dox. Rb indicates respiratory bronchiole. **c** Representative images (Magnification: 20×, Scale bar: 100 µM) of tissue sections from the LSL p53-R172H, shp53, K-Ras, SPC-Cre mouse line showing apparently normal lung Alveolar Type II cells immunostained for p53 (arrows); gross lung image shows formation of lung tumor (shown by black arrows) in –Dox. AR indicates alveolar region. **d** Representative images of normal tissue (+Dox) and tumor sections (–Dox) from LSL p53-R172H, shp53, K-Ras, CCSP-Cre and LSL p53-R172H, shp53, K-Ras, SPC-Cre mouse lines stained with hematoxylin and eosin (H&E) (Magnification: 10×, Scale bar: 200 µM) and immunostained with p53 antibody (Magnification: 20×, Scale bar: 100 µM). Rb indicates respiratory bronchiole. AR indicates alveolar region. **e** Images of tumor sections from LSL p53-R172H, shp53, K-Ras, CCSP-Cre and LSL p53-R172H, shp53, K-Ras, SPC-Cre mouse lines immunostained with SPC (red) and CCSP (green) fluorescent antibodies. Nuclei have been labeled by DAPI (4′,6-diamidino-2-phenylindole) (Magnification: 40×, Scale bar: 50 µM). Arrows indicate SPC-positive cells. CCSP-labeled cells were rare, possibly due to cell type conversion. **f** Representative images (Magnification: 20×, Scale bar: 100 µM) of tumor sections from CCSP and SPC mouse lines stained for thyroid transcription factor 1 (TTF-1) showing tumors are adenocarcinoma in nature. Arrows indicate positively stained cells.

We have generated a mouse model that expresses GOF p53 (p53-R172H) and K-Ras (G12D) in lung Club cells by cross-breeding Lox-Stop-Lox (LSL) p53-R172H^13^ and LSL K-Ras (G12D)^30^ mice. The resultant mice [LSL-R172H, LSL-K-Ras (G12D)] were crossed with mice expressing Cre recombinase from the Club cell secretory protein (CCSP) promoter, which is active in Club cells (CCSP-Cre)^31^ to produce the final mouse model line [LSL-R172H, LSL-K-Ras (G12D), CCSP-Cre]. To study the role of GOF p53 in tumor formation, these animals were crossbred with mice (TRE CMV.p53 shRNA, CCSP-rtTA) that can express p53 shRNA from a promoter with a tetracycline responsive element (TRE) after Dox-induction specifically in lung Club cells^32^ due to co-expression of a transactivator (rtTA) from the CCSP promoter^33^. The resultant mice [LSL-R172H, LSL-K-Ras (G12D), CCSP-Cre, TRE p53shRNA, CCSP-rtTA] were either treated with Dox to induce p53 shRNA and suppress GOF p53-R172H expression, or sucrose (control) to allow expression of GOF p53 (Fig. 1a–c and Supplementary Fig. 2f). Figure 1b, c shows immunohistochemistry of lung sections from mice treated with Dox (or sucrose control) and whole lung images. Mutant p53 expression is significantly reduced upon Dox treatment, with no tumor formation.

Following a similar procedure, we developed another construct where mutant p53 and K-Ras would be expressed under control of the surfactant protein C (SPC) promoter^34^ (Fig. 1c). The SPC promoter induces gene expression in alveolar type II epithelial (ATII) cells^35^. Many different types of cells can give rise to human lung adenocarcinoma, including ATII cells^36^. Oncogene expression under both CCSP and SPC promoters can lead to lung adenocarcinoma formation^37–39^. Figure 1d shows normal tissue and tumor sections from three mouse lung tumors expressing mutant p53 (or not, in the case of p53 shRNA expressing cells, normal tissue) from the CCSP or SPC promoter. Figure 1e shows sections from three tumors arising from both the LSL p53-R172H, K-Ras, shp53, CCSP-Cre and LSL p53-R172H, K-Ras, shp53, SPC-Cre mouse lines. Sections were stained for both CCSP and SPC proteins. SPC-labeled cells are clearly seen, while CCSP-labeled cells were rare, possibly due to cell type conversion. Tumors from mice expressing mutant p53 in both CCSP and SPC cells were stained with an antibody to thyroid transcription factor 1 (TTF-1) to distinguish the tumors as adenocarcinomas^40^ (Fig. 1f).

The data in Table 1 show that GOF p53 expression is required for lung tumor formation in the CCSP mouse line in a one-year timeframe in the presence of K-Ras. Tumors, judged to be lung adenocarcinomas, appeared in mice with GOF p53 expression and did not appear in mice treated with Dox inducing p53 shRNA and knocking down GOF p53 expression (Fig. 1). Thus, lung tumor formation in these lung cancer models is dependent on expression of p53-R172H.

**Table 1:** Lung tumor formation by GOF p53 and K-Ras expression.

	GOF p53 alone	GOF p53 with K-Ras
*Club cell secretary protein (CCSP) mouse genotypes*		
LSL p53-R172H, shp53, CCSP-Cre (Sucrose, no shp53 expression)	0/12	12/12
LSL p53-R172H, shp53, CCSP-Cre (Dox, shp53 expression)	0/12	0/12
LSL p53-L25Q/W26S/R172H, shp53, CCSP-Cre (Sucrose, no shp53 expression)	0/12	1/12
LSL p53-L25Q/W26S/R172H, shp53, CCSP-Cre (Dox, shp53 expression)	0/12	0/12
*Surfactant protein C (SPC) mouse genotypes*		
LSL p53-R172H, shp53, SPC-Cre (Sucrose, no shp53 expression)	0/9	9/9
LSL p53-R172H, shp53, SPC-Cre (Dox, shp53 expression)	0/9	0/9
LSL p53-L25Q/W26S/R172H, shp53, SPC-Cre (Sucrose, no shp53 expression)	0/9	0/9
LSL p53-L25Q/W26S/R172H, shp53, SPC-Cre (Dox, shp53 expression)	0/9	0/9

Histological sections show fairly circumscribed neoplastic proliferation forming nodules. The most common histologic growth pattern is acinar followed by papillary and solid. The tumors ranged from moderately to poorly differentiated with focal areas of necrosis. The background alveolated lung parenchyma shows peribronchiolar lymphocytic infiltrates suggestive of small airway disease. Interestingly, abundance of intra-alveolar macrophages with abundant eosinophilic granular cytoplasm can also be seen. No associated pigment, giant cells or granulomas were identified.

### p53-L25Q/W26S/R172H mouse is impaired in lung tumor formation

It is not known whether transactivation by mutant p53 is required for in vivo spontaneous lung cancer formation induced by GOF p53. Therefore, we tested this in the lung cancer model system by generating a conditional p53-L25Q/W26S/R172H mouse (Supplementary Fig. 1a–d), which has lung-specific expression of the mutant protein when crossed with CCSP-Cre mice^31^ to remove the LSL cassette. To determine the contribution of transcriptional activation of GOF p53 in lung tumor formation, the p53 L25Q/W26S/R172H mouse model was constructed to express mutant K-Ras as well [LSL-L25Q/W26S/R172H, LSL K-Ras (G12D), CCSP-Cre] (Supplementary Fig. 1). Altogether, we have generated the mouse strains shown in Table 1. Similarly, we expressed GOF p53- L25Q/W26S/R172H, and K-Ras under control of the human SPC promoter (Table 1).

Supplementary Figure 1e, f depicts immunohistochemistry of lung sections showing mutant p53 expression in L25Q/W26S/R172H mice under CCSP and SPC promoters when p53 shRNA expression is induced by Dox (or sucrose control). Representative images (Magnification: 20×) of the CCSP mouse line showing lung Club cells immunostained for p53 are shown; +Dox shows a decrease in p53 expression indicated by reduced staining intensity. We aged CCSP L25Q/W26S/R172H for 1 year and SPC L25Q/W26S/R172H for 7 months; in both cases the L25Q/W26S/R172H mice were defective in generating lung tumors (1/12 animals for CCSP L25Q/W26S/R172H, and 0/9 for SPC L25Q/W26S/R172H). Gross lung images show tumor formation for R172H and no tumor for L25Q/W26S/R172H (Fig. 1b, c and Supplementary Fig. 1e, f, arrows). These observations demonstrate that GOF p53-mediated transactivation is a key property needed for oncogenic activity of mutant p53 (Table 1).

### Human p53-R273H TAD defects reduce oncogenicity of H1299 cells

**Fig. 2:**
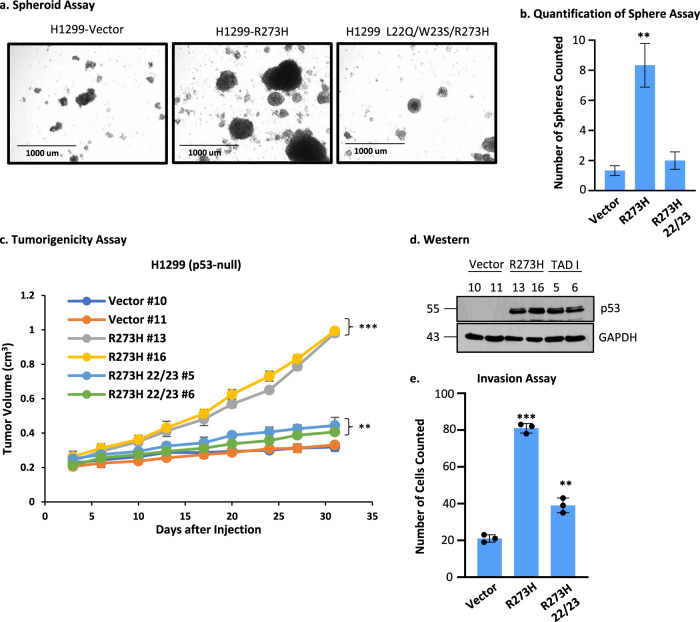
Human p53-R273H TAD defects reduce oncogenicity of H1299 cells. **a** Spheroid growth assay with H1299 p53-null lung cancer cells expressing p53-R273H, -L22Q/W23S/R273H, or vector control. Representative images are shown from each cohort. **b** Quantification of spheroid assay. The number of colonies were counted across multiple fields and plotted as the mean ± SEM (*n* = 3). A two-sided Student’s *t*-test was performed, **p = 0.0093. The most colonies are formed in H1299 p53-R273H cells. **c** A tumorigenicity assay was performed using cells from the spheroids formed in **a** injected subcutaneously in the flanks of NOD SCID mice. Data are presented as the mean ± SEM (*n* = 4). A two-sided Student’s *t*-test was performed comparing the vector cell lines, **p = 0.0369 and ***p = 0.00017. Vector control and L22Q/W23S/R273H grew much slower compared to R273H. **d** Western blot showing levels of p53 in cell clones. **e** Invasion assay performed using cells from the spheroids in **a**. Data are presented as the mean ± SEM (*n* = 3). A two-sided Student’s *t*-test was performed, **p = 0.0022 and ***p = 6.18E–6. Sphere formation, tumorigenicity, and invasion are all affected by mutation at 22/23.

To determine the impact of expressing a TAD mutant of the human p53-R273H in the human lung cancer cell line H1299 (p53 null) on tumorigenicity, we first isolated cancer stem-like cells as spheroids (Fig. 2a) in low serum media^41–43^ from H1299 cells stably transfected with vector alone, expressing p53-R273H, and p53-L22Q/W23S/R273H. Quantification of the spheroid assay indicated that the most colonies are formed in H1299 p53-R273H cells (Fig. 2b). Then, we injected identical numbers of cells from the spheroids into the flanks of nonobese diabetic/severe combined immunodeficiency (NOD SCID) mice. The data (Fig. 2c) show that vector control and L22Q/W23S/R273H grew much slower compared to R273H indicating that mutations in TAD significantly inhibited tumor growth. These data are consistent with the mouse lung cancer data presented above (Table 1). We also tested the invasion rate of these cells isolated from spheroids as measured by the migration of cells passing through Matrigel-coated membranes. The data shown in Fig. 2e demonstrate that mutations in TAD have a significant effect on invasion. Thus, sphere formation, tumorigenicity, and invasion are all affected by mutations at codons 22/23.

### Upregulation of many GOF p53 inducible genes requires an intact GOF p53 TAD

To determine if GOF p53-mediated gene upregulation requires an intact TAD, we generated H1299 cell clones expressing p53-R273H and the corresponding TAD mutant (L22Q/W23S/R273H), isolated independent cell clones, and then performed RNA sequencing (RNA-seq). Supplementary Data 1 shows examples of genes that require an intact TAD for induction with GOF p53. Supplementary Data 2 and 3 show the entire lists of genes that are activated by p53-R273H and require an intact TAD and that are activated by p53-R273H and can withstand mutations at positions 22/23, respectively. Western and RT-quantitative PCR (QPCR) data in Supplementary Fig. 2a, c show verification of some of the data shown in Supplementary Data 1. It is clear that many of the induced genes that are activated by p53-R273H need an intact TAD for optimal expression.

We also tested the level of expression of some of these genes in H1299 cells expressing a different GOF p53, p53-R175H (equivalent to murine p53-R172H), as well as its TAD mutant (L22Q/W23S; Supplementary Fig. 2d) and obtained data consistent with those found with p53-R273H and L22Q/W23S/R273H. We wanted to verify if similar genes are upregulated in mouse lung cells expressing the murine ortholog of human p53-R175H, p53-R172H, but not by p53-L25Q/W26S/R172H. Since only a few cells in the mouse lung express mutant p53 due to the way the mouse was constructed, we first generated cell lines homogenously expressing mutant p53 by harvesting lungs from p53‒/‒, p53+/+, p53 R172H/‒, and p53 L25Q/W26S/R172H/‒ mice and derived primary cultures from individual lung tissues. These cultures were infected with Adeno-Cre to release the LSL cassette (Supplementary Fig. 2e) so that most of the cells express mutant p53-R172H or L25Q/W26S/R172H. Supplementary Figure 2f shows a western blot demonstrating a robust expression of GOF p53 in mutant p53-expressing cells. Murine cells (p53‒/‒, p53+/+, p53 R172H/‒, and L25Q/W26S/R172H/‒) also show differences in spheroid formation and in invasion assays correlated with their p53 status (Supplementary Fig. 2g, i). We then tested the mRNA expression of multiple genes from Supplementary Data 1 by RT-QPCR. Supplementary Figure 2j shows data obtained using murine cells. The similarity of transcriptional data in Supplementary Fig. 2c, d, j, k suggests that, in general, TAD is required for GOF p53-mediated transactivation.

We have performed RNA seq on RNA from cells isolated from mouse lung and which are near-homogenously expressing p53‒/‒, p53-R172H/‒, and p53 R172H 25/26. The RNA seq data and its DAVID analysis is presented in Supplementary Data 5a, b.

### GOF p53 requires an intact TAD to get nucleated on promoter-enhancers of mutant p53 inducible genes

**Fig. 3:**
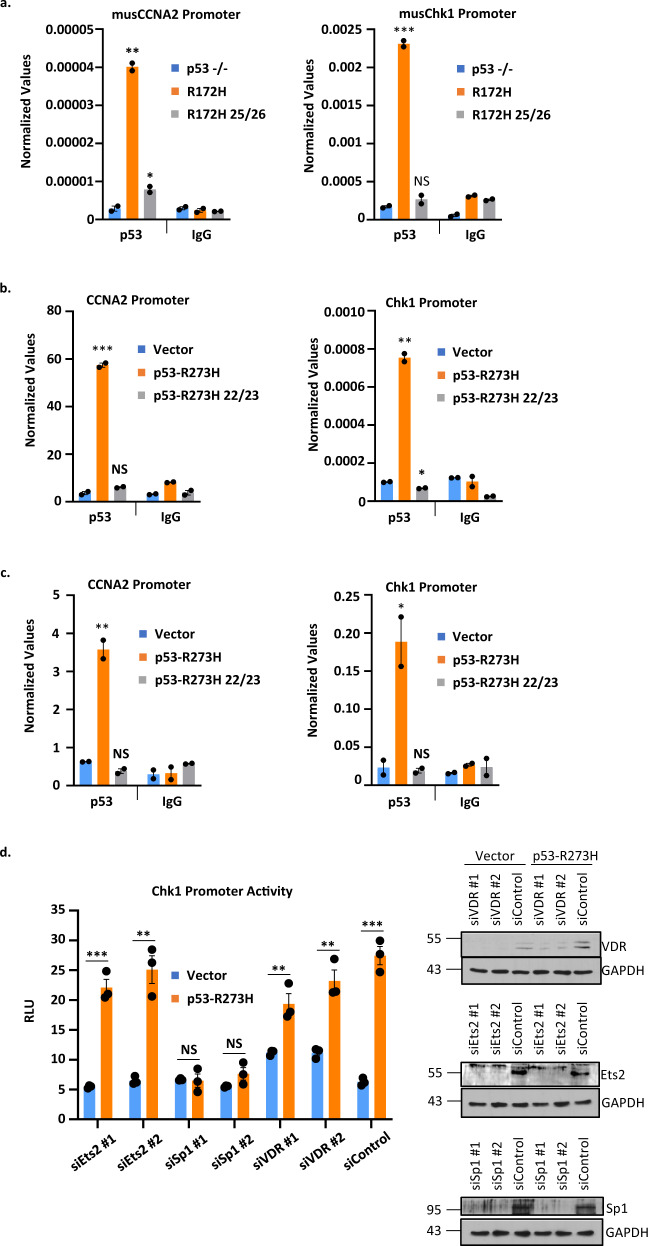
GOF p53 requires an intact TAD to get nucleated on upstream sequences of mutant p53 inducible genes in cells expressing mutant p53. Chromatin immunoprecipitation (ChIP) assays were performed to compare the ability of GOF p53 to interact on the promoter-enhancers of mutant p53-activated genes. **a** Mutant p53 ChIP was performed with murine cells with p53-R172H and p53-L25Q/W26S/R172H or p53–/– as a control. Mutations at 25/26 in GOF p53 impair the ability of the protein to anchor to the promoter as evident by the loss of ChIP. Mutant p53 ChIP was also performed with **b** H1299 cells expressing p53-R273H, -R273H L22Q/W23S, and vector control or **c** H1299 cells expressing p53-R175H, -R175H L22Q/W23S, and vector control. Data are normalized with input DNA values for each respective promoter and are presented as the mean ± SEM (*n* = 3). A two-sided Student’s *t*-test was performed; **a** *p = 0.038, **p = 0.001, ***p = 0.0003; **b** *p = 0.007, **p = 0.001, ***p = 0.0004; **c** *p = 0.038, **p = 0.0068. Mutations at 22/23 in human GOF p53 impair the ability of the protein to anchor to the promoter as evident by the loss of ChIP in **b** and **c**. **d** A transient transactivation assay was performed in H1299 using either pCMV Bam neo (Vector) or p53-R273H and siRNA against Ets2, Sp1, and VDR to show reduced transactivation of the Chk1 promoter by p53-R273H when Sp1 is knocked down. Data are presented as the mean ± SEM (*n* = 3, performed twice). *p-value < 0.05, **p-value < 0.01, and ***p-value < 0.001. NS no statistically significant difference from control.

Next, we investigated whether these mutations compromised the ability of GOF p53 to be nucleated on the promoter-enhancers of mutant p53 inducible genes. We performed chromatin immunoprecipitation (ChIP) assays in order to compare the ability of GOF p53 with intact or mutant TAD to interact on the promoter-enhancers of mutant p53 inducible genes. Figure 3a indicates the results of ChIP experiments demonstrating the inability of transactivation-deficient murine p53 mutant (L25Q/W26S/R172H, adeno-cre treated as described above), to nucleate on two promoters that we tested. The data suggest that GOF p53 needs an intact TAD in order to interact with, and transactivate, the promoter/enhancers. Similar assays were carried out for H1299 cells expressing p53-R273H, p53-L22Q/W23S/R273H, p53-R175H, and p53-L22Q/W23S/R175H with similar results (Fig. 3b, c). To determine the contribution of individual TFs in the induction of promoter activity by GOF p53, we have used siRNA against different TFs in H1299 cells stably transfected with p53-R273H and assayed promoter activity by luciferase assays. The data presented in Fig. 3d show that Chk1 promoter induction by p53-R273H is affected by Sp1 but not by VDR1 or Ets2 siRNAs, suggesting specificity attained by TF Sp1. These observations indicate an important role for TAD in the interaction of GOF p53 and its inducible promoter/enhancers.

### GOF p53 interacts with Med17 and amino acids at L25/W26 (mouse) and L22/W23 (human) are important for this interaction

Our data described above show that amino acids at positions 25/26 (mouse) and 22/23 (human) are crucial for GOF p53 nucleation on its inducible gene promoters and transactivation by GOF p53. Since GOF p53 seems to interact with DNA on the promoters through protein–protein interactions rather than through direct protein-DNA interactions^17,19^, we sought TFs that would interact with TAD at amino acids 25/26 for murine p53-R172H (22/23 for human). WT human p53 interacts with Mediator 17 (Med17) where interactions at amino acids 22/23 are crucial^24,44^. Therefore, we tested whether GOF p53-R172H and p53-L25Q/W26S/R172H as well as human p53-R273H, p53-L22Q/W23S/R273H, p53-R175H, and p53-L22Q/W23S/R175H interact with Med17, using immunoprecipitation (IP) and ChIP-re-ChIP approaches.

**Fig. 4:**
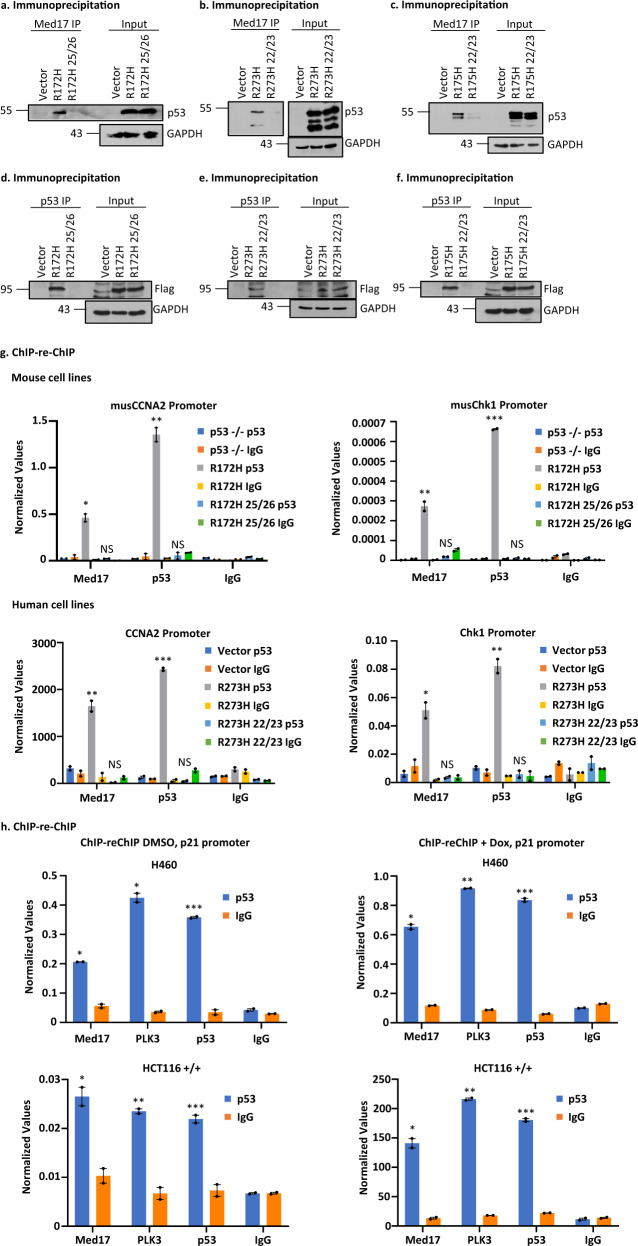
GOF p53 interacts with Med17 and amino acid acids at mouse p53 L25, W26 (L22, W23 in human) are important for this interaction. **a** Immunoprecipitation experiment showing complex formation between murine p53-R172H and the Mediator protein 17 (Med17) in solution. Nuclear extracts were made from murine lung cells expressing p53-R172H (generated by adeno Cre as described in the text), p53-L25Q/W26S/R172H, and control, and immunoprecipitated by Med17 antibody and probed with anti-p53 antibody. **b** Immunoprecipitation experiment showing complex formation between p53-R273H with Med17 in solution. Nuclear extracts were prepared from H1299 cells stably transfected with empty vector, expressing p53-R273H and -L22Q/W23S/R273H, and immunoprecipitated by Med17 antibody and probed with anti-p53 antibody. **c** Immunoprecipitation experiment showing complex formation between p53-R175H and Med17 in solution. Nuclear extracts were prepared from H1299 cells stably transfected with empty vector, expressing p53-R175H and -L22Q/W23S/R175H, and immunoprecipitated by Med17 antibody and probed with anti-p53 antibody. **d** Reverse immunoprecipitation experiment showing complex formation between murine p53-R172H and the Mediator protein 17 (Med17) in solution. Whole cell extracts were made from murine lung cells expressing p53-R172H (generated by adeno Cre as described in the text), p53-L25Q/W26S/R172H, and control after transfection with Flag-Med17, and immunoprecipitated by p53 antibody and probed with anti-Flag antibody. **e** Reverse immunoprecipitation experiment showing complex formation between p53-R273H and the Mediator protein 17 (Med17) in solution. Whole cell extracts were made from H1299 cells stably transfected with empty vector, expressing p53-R273H and -L22Q/W23S/R273H after transfection with Flag-Med17, and immunoprecipitated by p53 antibody and probed with anti-Flag antibody. **f** Reverse immunoprecipitation experiment showing complex formation between p53-R175H and the Mediator protein 17 (Med17) in solution. Whole cell extracts were made from H1299 cells stably transfected with empty vector, expressing p53-R175H and -L22Q/W23S/R175H after transfection with Flag-Med17, and immunoprecipitated by p53 antibody and probed with anti-Flag antibody. **g** ChIP-re-ChIP experiment showing complex formation on mutant p53 inducible gene promoters (cyclin A2 and chk1) between mutant p53 and Med17. ChIP-re-ChIP was carried out as described using murine lung cells described in **a** and H1299 cells described in **b**. The first immunoprecipitation was carried out with p53 antibody, while the second one was done using a Med17 antibody. Presence of promoter sequences was detected by QPCR using promoter specific primers. Data are normalized with input DNA values for each respective promoter and is presented as the mean ± SEM (*n* = 3). A two-sided Student’s *t*-test was performed; Mouse CCNA2 *p = 0.009, **p = 0.003, Mouse Chk1 **p = 0.007, ***p = 3.12E–5; Human CCNA2 **p = 0.008, ***p = 0.0002, Human Chk1 *p = 0.01, **p = 0.005. **h** ChIP-re-ChIP experiment showing complex formation on wild-type (WT) p53 inducible gene promoter p21 between WT p53 and Med17. ChIP-re-ChIP was carried out as described using H460 (WT p53) and HCT116 (p53+/+). The first immunoprecipitation was carried out with p53 antibody, while the second one was done using a Med17 antibody. Presence of promoter sequences was detected by QPCR using promoter specific primers. Data are normalized with input DNA values and is presented as the mean ± SEM (*n* = 3). A two-sided Student’s *t*-test was performed; H460 DMSO *p = 0.001, ***p = 0.0007; H460 Dox *p = 9.6E–4, **p = 1.05E–5, ***p = 2.29E–4; HCT116+/+ DMSO *p = 0.02, **p = 0.006, ***p = 0.009, HCT116+/+ Dox *p = 0.004, **p = 6.3E–5, ***p = 2.2E–4. Assay was performed with or without Dox treatment. NS no statistically significant difference from control.

As shown in Fig. 4a, Med17 and murine mutant p53-R172H co-immunoprecipitate when IPs were performed with a Med17 antibody and immunoblotted with p53 antibodies and this was significantly reduced upon mutation of p53 at amino acids L25Q/W26S. Similarly, we also tested whether TAD mutations affect the interaction of Med17 with human mutant p53-R273H and -R175H in H1299 cells and corresponding TAD mutant-expressing p53-L22Q/W23S/R273H and p53-L22Q/W23S/R175H cells (Fig. 4b, c). We have also tested the reverse IP by transfecting cells with Flag-Med17, pulling down the complex with p53 antibodies, and immunoblotting with Flag and found similar results. (Fig. 4d–f). In each case, the data are consistent with a role for amino acids 25/26 (mouse) and 22/23 (human) in the interaction of mutant p53 with Med17.

We also carried out ChIP-re-ChIP experiments, in which the initial ChIP was performed using a p53 antibody and the second IP was carried out using Med17 or p53 antibodies (or IgG as control). Data from these experiments are depicted in Fig. 4g, and show that the interaction between Med17 and mutant p53 is disrupted by TAD mutations for both murine p53-R172H and human p53-R273H, supporting our prediction. We have also studied the interaction of WT p53 and Med17 on the WT p53 target p21 promoter by performing similar ChIP-re-ChIP experiments. The data presented in Fig. 4h show interactions between Med17 and WT p53 on the promoter. Taken together, our data show that p53 and Med17 exist in a complex on WT- and mutant p53-inducible promoters.

### Med17 is crucial for mutant p53-mediated transactivation

It seems that GOF p53 interacts with Med17 and the interaction requires unchanged amino acids at positions 22/23 (human). Since this interaction correlates with GOF p53-mediated transactivation and nucleation of GOF p53, we hypothesized that at least one of the ways GOF p53 nucleation on a promoter happens is via its interaction with Med17. To test this hypothesis, we constructed WT p53-Med17, p53-R273H-Med17, and p53-L22Q/W23S/R273H-Med17 fusion constructs.

### Fusion of Med17 with TAD-mutated GOF p53 regenerates transactivation function

**Fig. 5:**
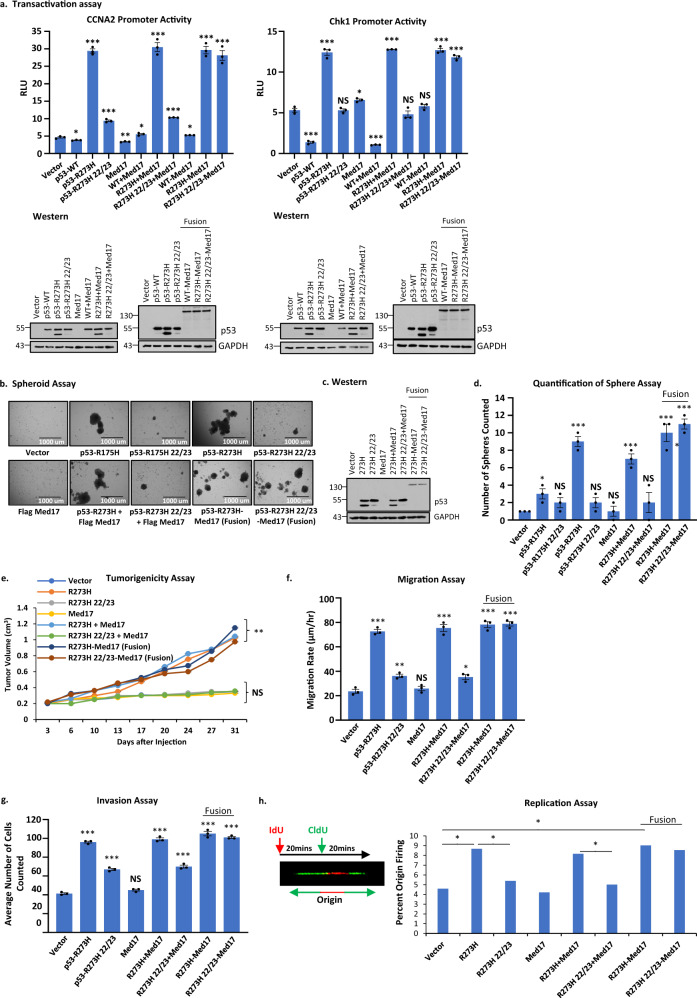
Fusion of Med17 with GOF p53 with TAD mutations regenerates transactivation and oncogenic activities. **a** Transactivation assay using GOF p53 inducible gene promoters and GOF p53 derivatives as indicated in the figure. Transient transcriptional assays were carried out in H1299 cells where transfections were carried out with empty vector or expression plasmid for indicated proteins. Cells were harvested after 24 h of transfection. Firefly luciferase readings were plotted as relative light units (RLU). Data are presented as the mean ± SEM (*n* = 3, performed twice). A two-sided Student’s *t*-test was performed. The data clearly show that transactivation by GOF p53 is lost by TAD mutation and is regained by fusion of the mutated GOF p53 with Med17. Western blots are shown below to indicate the level of expression of different proteins after transfection. Asterisks indicate a *p*-value < 0.001. **b** Spheroid growth assay using H1299 p53-null lung cancer cells expressing p53-R175H, -L22Q/W23S/R175H, -R273H, -L22Q/W23S/R273H, Med17, p53-R273H + Med17, p53-22/23/R273H + Med17, p53-R273H-Med17 (fusion), p53-22/23/R273H-Med17 (fusion), or vector control. Best colonies are formed in H1299 p53-R273H cells. **c** Western blot analysis of cells used for assays **b**–**h**. **d** Quantification of the Spheroid assay in **b**. The number of spheroids were counted in multiple fields and plotted as the mean ± SEM (*n* = 3). A two-sided Student’s *t*-test was performed. Colony formation ability is compromised by TAD mutations and is regained by fusion with Med17. **e** A tumorigenicity assay was performed using cells from spheroids in **b** injected subcutaneously in the flanks of SCID mice. Data are presented as the mean ± SEM (*n* = 4). An ANOVA was performed and **p = 0.003. The tumor-forming ability of L22Q/W23S/R273H was rescued with the fusion to Med17. **f** Migration assay using cells from the spheroids in **b**. Data are presented as the mean ± SEM (*n* = 3). A two-sided Student’s *t*-test was performed and *p = 0.01, **p = 0.004, and ***p < 0.0001. **g** Invasion assay using cells from the spheroids in **b**. Data are presented as the mean ± SEM (*n* = 3). A two-sided Student’s *t*-test was performed. **h** DNA replication origin firing assay using cells from the spheroids in **b**. Data are presented as the mean ± SEM (*n* = 200 DNA fibers). A two-sided Student’s *t*-test was performed. A cartoon is shown to depict the replication assay experimental scheme along with a representative image of a DNA fiber showing a fired origin. Migration, invasion, and origin firing abilities of L22Q/W23S/R273H were rescued with the fusion to Med17. *p-value < 0.05, **p-value < 0.01, and ***p-value < 0.001. NS no statistically significant difference from control.

We then carried out transcriptional transactivation assays as shown in Fig. 5a, using the cyclin A2 and Chk1 promoters driving luciferase expression (CCNA2-luc and Chk1-luc) as reporter constructs in p53-null H1299 lung cancer cells. The data show that GOF p53-R273H induced promoter activity as expected, and that this activation is lost in p53-L22Q/W23S/R273H. However, the activation is more than compensated when we used the p53-L22Q/W23S/R273H-Med17 fusion. This compensation is, however, not achieved when Med17 is expressed in *trans*. These data indicate that, for successful transactivation by GOF p53, Med17 should be in very close proximity with mutant p53, suggesting the interaction between Med17 and GOF p53 is crucial for transactivation by GOF p53.

After observing that fusion of Med17 can rescue the transactivation function of GOF p53 even with TAD mutations, we tested whether p53-L22Q/W23S/R273H-Med17 expressing cells could match p53-R273H in its oncogenic activities by generating H1299 cell clones expressing different derivatives, as indicated in Fig. 5b–h. We performed multiple growth-related assays: spheroid formation, tumor formation, motility and invasion assays, as well as an origin firing assay indicative of DNA replication capacity^45^. The data indicate that amino acid mutations at positions 22/23 have profound effects on GOF p53 oncogenic functions (Fig. 5b–h). The functional activity that was lost by TAD mutations was regained by fusion of mutant p53-L22Q/W23S/R273H with Med17. Therefore, the interaction of Med17 and mutant p53 is crucial for GOF p53 function and is important for recruiting mutant p53 near to the site where Med17 acts.

### TAD mutations affect the state of phosphorylation of mutant p53

**Fig. 6:**
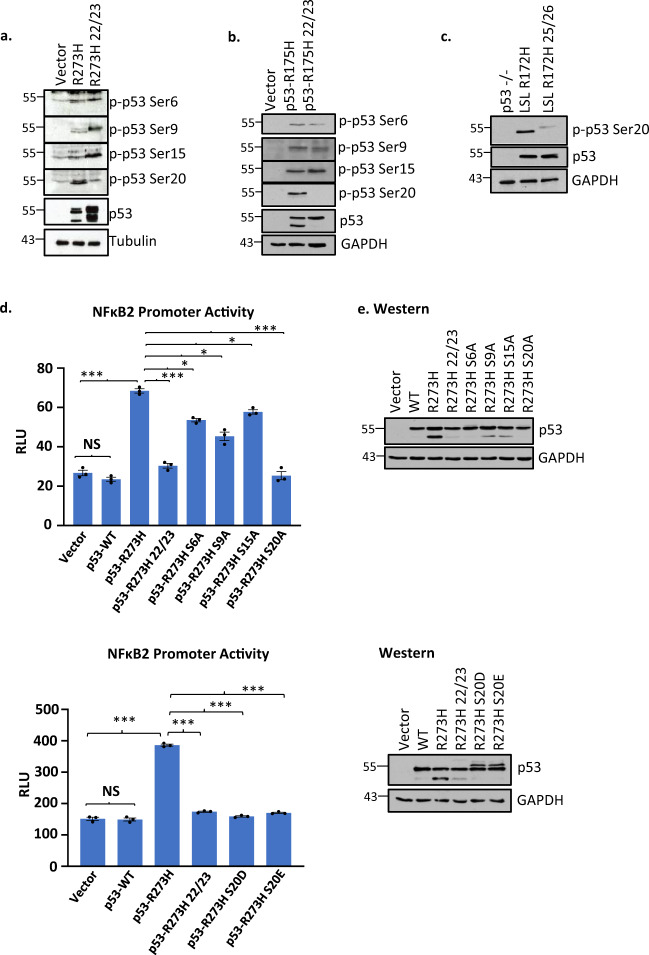
TAD mutations affect the state of phosphorylation of mutant p53. Western blot analysis of equal amounts of extracts from **a** H1299 cells expressing p53-R273H, -L22Q/W23S/R273H, and vector control; **b** H1299 cells expressing p53-R175H, -L22Q/W23S/R175H, and vector control; and **c** murine lung cells expressing p53-R172H, -L22Q/W23S/R172H, and p53–/– control were developed with antibodies against different phosphorylation sites on p53. The data presented show that phosphorylation at amino acid position 20 went down significantly. **d** Transactivation assays of mutant p53 and its derivatives by Ala substitution mutations at positions of phosphorylation using a mutant p53 inducible gene promoter. Transient transcriptional assays were carried out in H1299 cells after transfection of NF-κB2-luc and indicated expression plasmid or empty vector. Cells were harvested after 24 h of transfection. Firefly luciferase readings were plotted as relative light units (RLU). Data are presented as the mean ± SEM (*n* = 3, performed twice). A two-sided Student’s *t*-test was performed. Ser20Ala substitution reduced the transactivation ability the greatest. Substitution of S20 to Glu or Asp did not regain transactivation ability either. **e** Western blot analysis showing level of different p53 mutants after transfection. *p-value < 0.05, **p-value < 0.01, and ***p-value < 0.001. NS no statistically significant difference from control.

The TAD domain of p53 is activated by phosphorylation^29^. Thus, we assessed the difference in the phosphorylation status between p53-R273H and p53-R273H L22Q/W23S in H1299 cells to try to determine if an altered state of phosphorylation for the TAD mutant might contribute to a functional difference^46^. Figure 6a shows the results of western blot analysis of protein extracts from H1299 cells stably transfected with mutant p53-R273H and p53-L22Q/W23S/R273H, and developed with antibodies that recognize different phosphorylation sites on p53. Phosphorylation at amino acid position 20 was significantly reduced in p53-L22Q/W23S/R273H, whereas phosphorylation at other positions such as S9 and S15 increased inconsistently when amino acids at positions 22/23 (or mouse 25/26) were mutated. This suggests that post-translational modifications at specific amino acids may be important for GOF p53-mediated transactivation and are affected by mutations in nearby amino acids. The reason for lower S20 phosphorylation in p53-L22Q/W23S/R273H-expressing cells is not clear; one can conjecture that possible lower levels of PLK3 and/or steric issues may be the reason. Similarly, we tested if TAD mutations affect the state of phosphorylation of mutant p53 (R175H) in H1299 cells expressing p53-L22Q/W23S/R175H and in mouse lung cells expressing p53-L25Q/W26S/R172H compared to the clones expressing the mutants with intact TAD (Fig. 6b, c) and obtained similar data showing S20 phosphorylation is affected by TAD mutations.

To test whether phosphorylation at different amino acid positions is required for GOF p53-mediated transactivation, we generated a number of alanine (Ala) substitution mutants (S6A, S9A, S15A, and S20A). Figure 6d shows NF-κB2 promoter transactivation data obtained by co-expression of different Ala substitution mutants of p53-R273H in H1299 p53-null lung cancer cells. We found that S20A substitution has the most profound effect in reducing the transactivation ability, suggesting that phosphorylation at amino acid position 20 is important for transactivation by mutant p53. If the purpose of phosphorylation is merely to increase the negative charge of GOF p53, it would be predicted that substitution of S20 with the negatively charged glutamate (Glu) or aspartate (Asp) would still be capable of transactivation. Therefore, we made p53-R273H/S20D and /S20E and tested their transactivation capability. As shown in Fig. 6d, p53-R273H/S20D and p53-R273H/S20E failed to transactivate, thus a negative charge is not necessarily what is important. We are not sure why we are seeing a double band in the R273H/S20D and R273H/S20E western lanes; it is possible that excess negative charge is responsible for slower moving bands.

### PLK3 phosphorylates S20 of mutant p53 without induced DNA damage

**Fig. 7:**
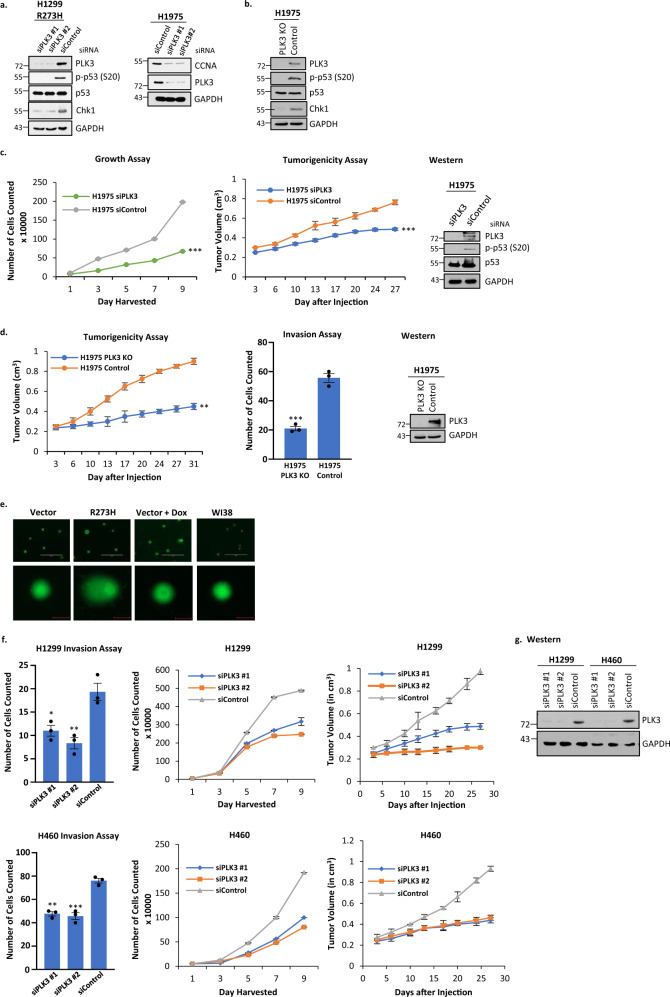
PLK3 phosphorylates S20 of mutant p53 and affects cell growth, tumorigenicity, and invasion in cells harboring GOF mutant p53. **a** H1299 cells expressing p53-R273H were transfected with siRNA specific for PLK3 (or scrambled control) and after incubation cells were harvested to analyze PLK3 levels and phosphorylation at S20 of p53. Data presented show that treatment of H1299 cells expressing p53-R273H with siPLK3 decreases the PLK3 protein concordant with loss of phosphorylation at S20; concomitantly the level of Chk1, a mutant p53 inducible gene, was reduced. An additional mutant p53 inducible gene, CCNA, was also reduced after PLK3 reduction in H1975. These observations suggest that S20 phosphorylation modulates transactivation of mutant p53. **b** A PLK3 knockout (PLK3 KO) cell line was generated to further prove that PLK3 modulates mutant p53’s transactivation via phosphorylation of S20. PLK3 was knocked out in the H1975 lung cancer cell line using CRISPR (region of deletion located to Chr1 44803749-44805264). Cell extracts from H1975 PLK3 KO and corresponding CRISPR control (Control) were harvested to analyze PLK3, p-p53 (S20), and Chk1 levels as shown. Data recapitulated those seen in the siRNA experiment shown in **a**. **c** H1975 lung cancer cells were transfected with siRNA against PLK3 (or scrambled control) and growth and tumorigenicity assays were performed. Data are presented as the mean ± SEM (*n* = 3 for growth assay, *n* = 4 for tumorigenicity assay). A two-sided Student’s *t*-test was performed; Growth assay ***p = 1.8E–7, Tumorigenicity assay ***p = 5.2E–5. In both assays, siPLK3 reduced the oncogenic potential of the cell line. **d** A PLK3 knockout (KO) cell line was generated to further prove that PLK3 can be targeted to reduce the oncogenicity of lung cancer cells harboring mutant p53. PLK3 was knocked out in the H1975 lung cancer cell line using CRISPR (region of deletion located to Chr1 44803749-44805264) and tumorigenicity and invasion assays were performed. Data are presented as the mean ± SEM (*n* = 4 for tumorigenicity assay, *n* = 3 for growth assay). A two-sided Student’s *t*-test was performed; Invasion assay ***p = 0.0004, Tumorigenicity assay **p = 0.007. Both assays showed that the tumorigenic and invasive properties are severely reduced when PLK3 is knocked out. Western blots show levels of PLK3/p53/phospho-p53. **e** A comet assay was performed on H1299 cells stably transfected with empty vector and expressing p53-R273H. H1299 cells stably transfected with empty vector and treated with doxorubicin were used as a positive control while untreated WI38 cells were a negative control. (Scale bar: 400 µM, top row; 40 µM bottom row). **f** Invasion, growth, and tumorigenicity assays were performed on H1299 and H460 cells after PLK3 was knocked down using siRNA. Data are presented as the mean ± SEM (*n* = 3 for invasion and growth assays, *n* = 4 for tumorigenicity assay). **g** Western blot analysis of cells used in **f** showing efficient knockdown of PLK3 after siRNA treatment. *p-value < 0.05, **p-value < 0.01, and ***p-value < 0.001. NS no statistically significant difference from control.

PLK3 has been reported to phosphorylate WT p53 at S20 under conditions of DNA damage^27,47^. We found that PLK3 was upregulated in H1299 cells stably expressing p53-R273H versus cells transfected with vector control, and that this upregulation is reduced when the TAD is mutated at 22/23 (Supplementary Fig. 3a). In addition, phosphorylation of a PLK3 substrate, TOP2A, is also reduced in H1299 cells stably expressing L22Q/W23S/R273H (Supplementary Fig. 3b). Since expressions of PLK3 and phosphorylation of p53 are both reduced when the TAD of mutant p53 is altered, we wanted to determine if inhibiting PLK3 would impact S20 phosphorylation of mutant p53. Figure 7a shows that treatment of H1299 cells expressing p53-R273H together with PLK3 siRNAs or control shows reduction in the level of PLK3 protein, concomitant with a loss of phosphorylation at S20 of p53. We also tested a mutant p53-inducible gene product, Chk1, to determine if the level of the protein is modulated by inhibiting S20 phosphorylation. H1975 cells (endogenous R273H expression) were used to show that reduction of another mutant p53 inducible gene, cyclin A, also occurred after PLK3 siRNA treatment. The data shown in Fig. 7a indicate that reduction of S20 phosphorylation is accompanied by decreased Chk1 expression, consistent with reduced transactivation activity of GOF p53.

A PLK3 knockout (PLK3 KO) cell line was generated from H1975 cells to further test if PLK3 modulates mutant p53’s transactivation via phosphorylation of S20. PLK3 was knocked out in the H1975 lung cancer cell line using CRISPR. Cell extracts from H1975 PLK3 KO and corresponding CRISPR control were harvested to analyze PLK3, p-p53 (S20), and Chk1 levels as shown. Data shown in Fig. 7b recapitulated those seen in the siRNA experiment shown in Fig. 7a. Thus, PLK3 regulates phosphorylation of p53 at S20 and transactivation by GOF p53 without induced DNA damage.

### PLK3 promotes growth and tumorigenicity of lung cancer cells expressing GOF p53

PLK3 has been shown to phosphorylate S20 on WT p53 and promote apoptosis^27,47^, but we predicted that PLK3-mediated phosphorylation of S20 of GOF p53 would do the opposite and promote cell growth and tumorigenicity. To test if PLK3 might be used as a target to inhibit lung tumor growth, we used PLK3 siRNA to reduce PLK3 levels and determined the effect on growth and tumorigenicity of H1975 cells. Data presented in Fig. 7c indicate that lowering PLK3 levels reduces the rate of cell growth in culture as well as tumor formation in NOD SCID mice. H1975 PLK3 knockout cells gave similar results in tumorigenicity and invasion assays (Fig. 7d). Conversely, overexpression of PLK3 in H1299 cells stably transfected with vector control or expressing p53-R273H with and without the presence of Kras G12D showed an increase in functional assays such as invasion and growth without inducing apoptosis (Supplementary Fig. 4). Thus, PLK3 acts as a pro-oncogenic factor in cooperation with tumor-derived mutant p53 in contrast with WT p53, whose tumor suppressor activity it supports. These data suggest that PLK3 may be a possible therapeutic target for lung cancer harboring GOF p53 mutations.

As shown in Fig. 7e, we have performed a comet assay to determine if induced DNA damage can be detected in our cells. As predicted, we observed DNA damage only in cells treated with a DNA damaging agent. We then asked if PLK3 could affect oncogenicity in the absence of GOF p53. We answered this by using H460 (WT p53+/+) and H1299 (p53‒/‒) cells and studying their growth rate, invasion and tumorigenicity in nude mice. As shown in Fig. 7f, g, PLK3 knockdown affects all the three properties irrespective of the presence of p53. This suggests that mutant p53 enhances the pro-oncogenic influence of PLK3 by using it in its oncogenic pathway.

### PLK3 controls transactivation by GOF p53

**Fig. 8:**
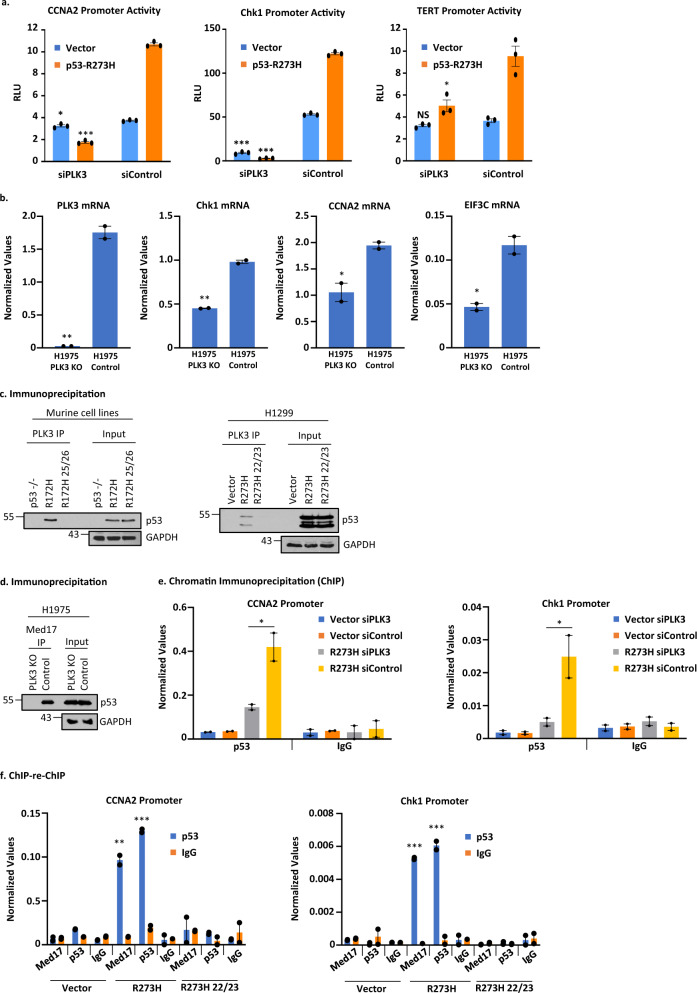
PLK3 controls GOF p53’s transactivation and binding to its inducible gene promoters. **a** Transient transactivation assay of H1299 cells transfected with the p53-R273H (or empty vector) along with siRNA against PLK3 (or scrambled control) and the cyclin A promoter, Chk1 promoter, or the TERT promoter-luciferase construct. Transient transcriptional assays were carried out in H1299 cells. Cells were harvested after 24 h of transfection. Firefly luciferase readings were plotted as relative light units (RLU) and is presented as the mean ± SEM (*n* = 3, performed twice). A two-sided Student’s *t*-test was performed and CCNA2 *p = 0.01, ***p = 6.5E–7; Chk1 ***p < 2.5E–6; TERT *p = 0.01. **b** H1975 PLK3 KO cells were used to determine the ability of GOF p53 to upregulate its activated genes. Transcript levels of PLK3, Chk1, CCNA2, and EIF3C were measured by QPCR. Data are normalized with GAPDH and are presented as the mean ± SEM (*n* = 3). A two-sided Student’s *t*-test was performed and PLK3 **p = 0.002; Chk1 **p = 0.001; CCNA2 *p = 0.03; EIF3C *p = 0.02. **c** Co-immunoprecipitation of mutant p53 and PLK3 in murine and human cells. Mouse R172H and human R273H were able to interact with PLK3 but their transactivation-deficient counterparts, L25Q/W26S/R172H and L22Q/W23S/R273H were not. **d** PLK3 knockout (KO) cells were used to determine whether the interaction between mutant p53 and Med17 has any requirement for PLK3. Co-immunoprecipitation in H1975 PLK3 KO cells versus control showed that the interaction between mutant p53 and Med17 is lost when PLK3 is knocked out. **e** The ability of mutant p53 to bind to its inducible promoters was determined by performing a ChIP assay. H1299 cells expressing p53-R273H (or containing empty vector) were transfected with siRNA against PLK3 (or scrambled control) and p53 ChIP was performed. Data are normalized with input DNA values for each respective promoter and is presented as the mean ± SEM (*n* = 3). A two-sided Student’s *t*-test was performed and *p = 0.02. **f** ChIP-reChIP assays were performed to determine if the complex between PLK3 (and/or Med17) and mutant p53 are present on mutant p53’s inducible promoters using H1299 cells stably expressing the p53-R273H or -L22Q/W23S/R273H (or containing empty vector). The first immunoprecipitation was carried out with Med17 or PLK3 antibodies while the second IP was done using a p53 antibody (or IgG control). The presence of promoter sequences was detected by QPCR using promoter specific primers. Data are normalized with input DNA values for each respective promoter and are presented as the mean ± SEM (*n* = 3). A two-sided Student’s *t*-test was performed and *p-value < 0.05, **p-value < 0.01, and ***p-value < 0.001. NS no statistically significant difference from control.

p53 is phosphorylated at S20 by PLK3 and Chk2 following ionizing radiation^29,48^. Since PLK3 controls phosphorylation at S20, and S20 phosphorylation is affected by mutations at amino acids 22/23, we tested if PLK3 could influence transcriptional induction by GOF p53. Thus, we used PLK3 siRNA to reduce the PLK3 level and then measured promoter activation by GOF p53. Data shown in Fig. 8a demonstrate that reducing the PLK3 level lowers transactivation by GOF p53 as shown by transient transcriptional assays using H1299 lung cancer cells and different promoter-luciferase constructs whose promoter activities were inhibitied by PLK3 siRNA (Fig. 8a). mRNA levels measured by QPCR from PLK3 knockout cells compared to control cells corroborate these data for several GOF p53 inducible genes (Chk1, CCNA2 and EIF3C, Fig. 8b).

### GOF p53 interacts with PLK3 and amino acids L25/W26 (mouse) and L22/W23 (human) are important for this interaction

As described above, PLK3 controls phosphorylation at S20 in GOF p53. Therefore, we tested whether PLK3 forms a complex with GOF p53 and, if so, whether that interaction would be lost upon mutation at 25/26 (murine) or 22/23 (human). We then performed experiments where we immunoprecipitated PLK3 and determined the presence of p53 using murine lung cells expressing p53-R172H or its 25/26 mutant and human H1299 lung cancer cells expressing p53-R273H or its 22/23 mutant. Figure 8c shows that murine R172H and human R273H form a complex with PLK3 and that this interaction is lost when TAD is mutated in each system.

### Med17 is inhibited in its interaction with GOF p53 in PLK3 knockout H1975 cells

Since PLK3 controls S20 phosphorylation, we tested if absence of PLK3 would impact S20 phosphorylation and GOF p53’s interaction with Med17, an interaction that we found to be necessary for transactivation. Therefore, we tested the in vivo interactions between Med17 and GOF p53-R273H in H1975 control and PLK3 knockout cells by performing co-IPs. Data in Fig. 8d show that while in control cells there is a clear interaction between Med17 and p53-R273H by co-IP, the interaction is drastically reduced in the PLK3 knockout cell clone.

### PLK3 assists in GOF p53’s interaction with mutant p53 inducible gene promoters

Lowering the PLK3 level inhibited GOF p53-mediated transactivation. Therefore, using H1299 cells stably transfected with empty vector or expressing p53-R273H (both lines also transfected with PLK3 siRNA or control siRNA), we next tested if depletion of PLK3 would cause disruption of the interaction between GOF p53 and target promoters by performing anti-p53 ChIP. Data presented in Fig. 8e show that in H1299 cells expressing p53-R273H, nucleation of mutant p53 on mutant p53-inducible promoters, as assayed by ChIP, decreased after PLK3 siRNA treatment for both the CCNA2 and Chk1 promoters. Thus, PLK3 aids the nucleation of mutant p53 on its target promoters, presumably by regulating S20 phosphorylation.

### PLK3 is located on mutant p53 inducible promoters complexed with mutant p53

Since PLK3 seems to assist in GOF p53’s interaction with its inducible promoters, we determined whether it is localized on mutant p53 inducible promoters. ChIP-re-ChIP experiments were performed using H1299 cells stably transfected with empty vector or expressing p53-R273H (or p53-L22Q/W23S/R273H) where the first IP was done using an antibody against PLK3 and the second IP was performed using a p53 antibody. Data presented in Fig. 8f show that, for both promoters tested, we could demonstrate an interaction between p53-R273H and PLK3. This was also seen with the interaction between Med17 and GOF p53 where the first IP was done using an antibody against Med17 and the second IP was performed using a p53 antibody. These interactions are compromised by mutations of amino acids 22/23 of GOF p53. To determine if the p53-PLK3 interaction on a mutant p53 inducible gene promoter is induced under conditions of stress, we carried out ChIP-re-ChIP in H1299 cells stably transfected with empty vector or expressing p53-R273H after doxorubicin treatment and saw an increase in PLK3 binding after Dox treatment (Supplementary Fig. 3c). Thus, PLK3 and GOF p53, and Med17 and GOF p53, operate in close proximity to each other in transactivating a GOF p53 inducible gene.

## Discussion

We report the generation of a mouse lung adenocarcinoma model in which tumorigenesis is dependent on the presence of GOF p53 and, more specifically, GOF p53’s transcriptional activation ability (Table 1). Expressing GOF p53 and activated K-Ras in Club or alveolar cells generated similar results. The dependence of tumorigenicity on the presence of GOF p53 in mouse models is reminiscent of GOF p53 expressing cancer cells’ dependence on GOF p53 for its oncogenicity in cell culture models^12,20,49,50^. Thus, this mouse model can be used to study different molecular and cellular aspects of mutant p53 dependency.

The concept of GOF p53-dependent oncogenesis relies on many pieces of evidence collected over years, including generation of knock-in mice that suggest that the presence of mutant p53 endows cancer cells with an extra oncogenic advantage^2,4,17^. This is backed by identification of mutant p53 inducible genes and identification of mutant p53 interaction sequences on the target promoters by several laboratories^20,22,51–54^. However, more recently using myeloid malignancies, Boettcher et al.^55^ could not find GOF activity in their system with the p53 mutants tested; they could explain their oncogenic data by a dominant negative effect of mutant p53. As suggested by them, the difference between their results and others could be dependent on cellular context, the type of cancer, and the concentration of various TFs present in the cell.

Our work using these models is an in vivo demonstration that GOF p53 requires its transactivation function to induce cancer (lung adenocarcinoma) in mice. This was established using a transactivation-deficient mutant p53-L25Q/W26S/R172H, which in general failed to produce tumors in mouse lung (Table 1). Our mouse model data have been corroborated by xenograft data generated in immunodeficient mice with human lung cancer cell lines either expressing GOF p53 or its transactivation-deficient (L22Q/W23S) derivative (Fig. 2c). Many genes induced by GOF p53 require an intact TAD (Supplementary Data 1, 2, and 5a, b). The fact that mutations at amino acids 22/23 in human mutant p53 disrupt both transactivation and tumorigenesis, an observation recapitulated in the murine system, strongly suggests that, for GOF p53 induced lung oncogenesis, the pro-oncogenic targets that are activated by GOF p53 are valuable for functional studies. Indeed, RNA-seq analysis of cell lines derived from our R172H and transactivation-deficient mutant p53-L25Q/W26S/R172H mice showed that multiple signaling pathways are affected by mutations within the TAD and could account, at least partially, for the inability for that mouse to produce lung tumors. This agrees with published works^2,17,19^. Along with GOF p53 itself, gene products inducible by mutant p53 can also be efficient targets for therapy^45^.

We determined that the murine GOF p53 TAD mutant L25Q/W26S causes the protein to fail to interact with its inducible gene promoters (Fig. 3), suggesting that anchoring of GOF p53 on the promoter depends on factors that interact with the TAD. Mutations at amino acids 25/26 (murine) or 22/23 (human) cause inhibition of interaction with Med17 (Fig. 4). This observation is in agreement with the property of WT p53 interacting with Med17^24,44^. If Med17 recruits GOF p53 on the promoter, and that nucleation is lost as a result of TAD mutations, we reasoned a fusion between Med17 and GOF p53 with mutated TAD would restore the transactivation activity, which was indeed observed (Fig. 5a). Our data suggest that in the absence of Med17-GOF p53 interactions mutant p53 cannot be on the promoter even though TFs are on the DNA. We propose that the TF-mutant p53 interaction site is also needed.

**Fig. 9:**
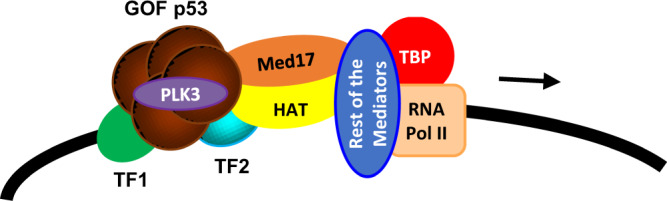
Proposed model for GOF p53 nucleating on an inducible promoter. Arrow toward the right-hand side depicts transcription direction. For simplicity TAF and other factors are not shown.

It was speculated previously that GOF p53 may nucleate on its target promoters-enhancers via a TF that has a site on the promoter-enhancer^17,19^. The Mediator complex is, in general, believed to act as a bridge between sequence-specific TFs that are gene specific and the transcription complex formed by RNA polymerase II^23,44,56,57^. Thus, it is possible that in the presence of GOF p53 the Mediator complex interacts with Sp1 or other TFs as well as the bound GOF p53 on the TFs (see Fig. 9). Although not all promoters have Sp1 binding sites, we believe mutant p53 has the ability to bind to two same or different TFs positioned appropriately on a promoter^51,58^. This dual interaction may further regulate transactivation by GOF p53 and result in looping from distant sites on the enhancer^49^. Thus, the amino acids in close proximity to positions 22/23 of GOF p53 are believed to be involved in interactions with both Sp1 or other transactivators, as well as a component of the Mediator complex, Med17. Whether mutations of GOF p53 at positions 22/23 affect its interactions with other members of the Mediator complex is not known at this time.

Mutant p53-R273H interaction on genomic DNA has been analyzed by us previously^51^ using ChIP seq to identify sequence motifs responsible for interacting with mutant p53. Our work demonstrated that both ETS1 and GABPA showed an increase in the probability of binding to promoters with a higher number of ETS1 motifs. We proposed a model for the binding of ETS1 and p53 R273H in which two inverted ETS motifs on a looped DNA helix are juxtaposed for ETS1 binding as a homodimer, with p53 R273H bound to ETS1^51^.

Mutations at amino acids 22/23 of GOF p53 cause changes in post-translational modifications, i.e., phosphorylation (Fig. 6a–c). It is possible that conformational or steric changes related to mutations at positions 22/23 result in decreased access of some protein kinases that phosphorylate these sites leading to a decrease of phosphorylation. Alanine substitutions at the respective amino acids caused a significant reduction of activity at S20, suggesting phosphorylation at that position serves an important function in the mechanism of transactivation (Fig. 6d). The S20A mutant may fail to transactivate because of the absence of negatively charged amino acids. However, when we altered the sequence to encode aspartate or glutamate, which would endow it with a negative charge, transactivation capability was not restored (Fig. 6d). These data, combined with the involvement of PLK3 in transactivation by GOF p53 as discussed below, indicate that phosphorylation at S20 plays an important role in transactivation by GOF p53. It is possible that S20 phosphorylation may play some role in GOF p53’s possible interaction with p300/CBP^59,60^. We do not imply that our data indicate that mutant p53 acquires sequence-specific DNA-binding ability. Any sequence specificity that it acquires is likely via mutant p53’s interaction through TFs (TF1 and TF2 in Fig. 9).

We determined if PLK3 phosphorylates the serine residue at position 20 in the absence of obvious DNA damage (Fig. 7a, b). PLK3 has been shown to interact physically with p53 and phosphorylate it^27,47^. Depletion of PLK3 adversely affects functions of GOF p53 as it inhibits transactivation since GOF p53 can no longer interact efficiently with Med17 (Figs. 7 and 8d). Interestingly, transactivation of Chk1 did not exhibit mutant p53-PLK3 dependence after siPLK3 knockdown, showing that there are some promoters that are independent of the interaction between mutant p53 and PLK3 (Fig. 8a). PLK3 knockdown inhibits interactions of GOF p53 with its inducible gene promoters (Fig. 8e). Data in Fig. 8f show that both Med17 and PLK3 are present on mutant p53 inducible gene promoters in complex with GOF p53, raising the attractive possibility that PLK3 may phosphorylate GOF p53 at the transcription complex keeping the complex active. Removal of PLK3 thus lowers the functional capacity of the complex.

Data from Figs. 7 and 8 suggest that phosphorylation S20 of mutant p53 may enhance transactivation and oncogenic functions of mutant p53 via PLK3. However, it is clear that PLK3 has a GOF p53-independent role and GOF p53 has PLK3-independent roles, but both proteins together enhance oncogenesis significantly. We have also included our analysis of H1975 shp53 cells/shGFP cells and performed QPCR assays to measure mRNA levels of different mutant p53 inducible genes in PLK3 knockout cells (and controls). The data show a similar trend, as discussed above.

At this stage, we do not know if all mutant p53 interactions or gene transactivation by GOF p53 are modulated by PLK3. All these data suggest that, as outlined in the model shown in Fig. 9, PLK3 may be included as part of a complex to phosphorylate GOF p53, thus maintaining it in a transcriptionally active form. Therefore, PLK3 can have a dichotomous role in the cell depending on the p53 status. PLK3 has been shown to phosphorylate S20 on WT p53, activating the tumor suppressor function and promoting apoptosis^27,28^. Yet, our results show how PLK3 can perform the same action, phosphorylating S20 on GOF p53, which is no longer a tumor suppressor but an oncogene, and no longer pro-apoptotic but pro-proliferative and pro-tumorigenic. Thus, GOF p53 aids PLK3 in oncogenesis, raising the possibility of targeting GOF p53 inducible oncogenesis using PLK3 inhibitors.

## Methods

### Knock-in mouse construction

In general, the procedure was as described^61^. The targeting construct is shown in Supplementary Fig. 1a. G418-resistant embryonic stem (ES) clones were screened for homologous recombination by a combination of long-range PCR and Southern blot analysis of AflII digests. Sequencing of the entire open reading frame of the p53 gene and all intron/exon boundaries in the targeting vector was done to confirm the presence of the mutations and to verify the integrity of the p53 gene. The cloning vector was electroporated into 129/Sv ES cells. PCR and Southern hybridization analyses detected homologous recombinants (Supplementary Fig. 1b, c. Correctly targeted clones were injected into 129/Sv blastocysts, which were then transferred into pseudo-pregnant female recipients to generate chimeras. Germ-line transmission was observed (Supplementary Fig. 1), and sequencing of tail DNA showed the presence of the engineered mutations (Supplementary Fig. 1d).

### Mouse treatment

A total of 500 µg/mL doxorubicin in 5% w/v sucrose was used for treatment of mouse models.

### Cells

H1299 (p53-null) and H1975 (p53-R273H) cell lines were purchased from American Type Culture Collection, and were maintained in media as suggested by the suppliers. No additional authentication was performed on these cells. Methods for lipofection and generation of stable transfectants were as described earlier^11,62^. Clones were isolated using puromycin selection at 1 µg/mL. Transfections were performed using Lipofectamine 2000 (ThermoFisher) following the manufacturer’s instructions. Transient promoter transactivation assays were performed in triplicate using 1ug of empty vector or p53-R273H as well as 200 ng of promoter-luc. Murine cell lines were created by harvesting mouse lung and isolating lung cells from respective mice by using sterile scalpels to mince the tissue. Tissue pieces were allowed to adhere to culture dishes in DMEM, 10% FBS, and in the presence of penicillin/streptomycin and gentamicin. Cells that adhered to culture dishes were then cultured and used for experiments.

### Spheroid formation assay

Cells were plated in ultra-low attachment plates (Sigma) at a cell density of 5 × 10^4^ cells/well of a 24-well plate. The base medium used was DMEM:F12 supplemented with 4 μg/mL insulin, 20 ng/mL EGF, 20 ng/mL bFGF, and B27 at a final concentration of 2% as described^42,43^. Pictures were taken 1 week after plating and a representative image is given.

### H1299 cells expressing GOF p53 mutants

To determine the influence of TAD on mutant p53-mediated transactivation, we have constructed three amino acid substitution mutants p53-L22Q/W23S/R273H and -L22Q/W23S/R175H using the Quickchange mutagenesis kit (Agilent Technologies) and expressed these p53 mutants in H1299 cells. Several clones were isolated with expression equivalent to cells expressing p53-R273H alone. We used these clones in comparison with vector transfected cell clones for our assays. We have also used H1299 cells expressing p53-R175H and -R273H (or vector transfected cells) as described earlier^63^.

### Doxorubicin treatment

Doxorubicin treatment of cells in culture was done using a concentration of 2 µM for 24 h.

### CRISPR/Cas9 knockout of PLK3

To test how PLK3 gene elimination would affect oncogenic properties of lung cancer cells with GOF p53, we generated PLK3 knockout clones by CRISPR/Cas9 using gRNAs as described^64^. PLK3 gRNAs (BRDN0001146265, BRDN0001146656, and BRDN0001145739) were gifts from John Doench and David Root (Addgene plasmid # 77470, 77471, and 77472). We identified at least two knockout clones and used one of them in our assays.

### Growth assay

Growth assays were carried out as described by us earlier with slight modifications^5,63,65^. Cells were plated at 50,000 cells/6 cm dish in triplicate for five time points and harvested after incubation with trypsin and counted using a Coulter Counter (Beckman). Multiple cell clones were used for each assay. All experiments were done in triplicate, and repeated multiple times. Error bars represent standard deviation from the triplicates.

### Migration assays

Cell migration was carried out using wound-healing (scratch) assays, as previously described^66^. Briefly, cells were trypsinized, plated in quadruplicate in 12-well cell culture plates and incubated at 37 °C until cells were completely confluent. At this time, a sterile pipette tip was used to scratch across the surface of the plate, removing the complete layer of cells within the scratch area. Following cell removal, each well was washed once with PBS and then replaced with growth medium. Immediately following, the width of the scratch was measured at six specific points under a 5× objective using a light microscope and AxioVision software (Carl Zeiss Microimaging, Thornwood, NY). Cells were incubated at 37 °C from 20 to 60 h depending on the cell line under study, at which time the scratch width was measured at the same position as at time 0. Graph represents the average of triplicate values for each experiment.

### Invasion assays

Invasion assays were carried out as described^67^. Matrigel inserts were rehydrated in serum-free media at 37 °C for 2 h. Cells were counted and 0.5 mL of a 5 × 10^4^ cells/mL cell concentration was seeded on top of the matrix in triplicate. Media and chemoattractant were added to the lower chamber and plates were incubated at 37 °C for 24 h. Filters were treated as described and cells were counted. Graphs represent the average of triplicate values for each experiment. Error bars shown represent standard deviations.

### Tumorigenicity assay

Tumorigenicity assays were performed using either NOD SCID or Nu/J (nude) mice. A total of 1 × 10^7^ cells were used to inject into both flanks of two mice and tumors were measured by calipers and allowed to grow to a maximum size of 1 cm^3^. For tumorigenicity assays that were performed after transfection, cells were counted the day of injection (48–72 h post transfection).

### Comet assay

The comet assay was performed using the kit from Trevigen and following the manufacturer’s protocol.

### Western blotting

Immunoblotting was carried out as described^63^. Westerns blots were developed by the ECL method (GE Healthcare; Piscataway, NJ). Antibodies used for western analysis include: p53 (PAb 1801, 1:100), EIF3C (2068S, 1:1000, Cell Signaling), EGR1 (4154S, 1:1000, Cell Signaling), cyclin A (sc-239, 1:100, Santa Cruz), cyclin D2 (2924S, 1:200, Cell Signaling), GAPDH (sc-32233, 1:200, Santa Cruz), Tubulin (2148S, 1:200, Cell Signaling), p-p53 Ser6 (9285S, 1:1000, Cell Signaling), p-p53 Ser9 (9288S, 1:1000, Cell Signaling), p-p53 Ser15 (9286S, 1:1000, Cell Signaling), p-p53 Ser20 (9287S, 1:1000, Cell Signaling), PLK3 (4896S, 1:1000, Cell Signaling), Chk1 (2360S, 1:1000, Cell Signaling), Flag (F1804, 1:1000, Sigma Aldrich), PARP (9532, 1:1000, Cell Signaling), p-TOP2a (PA5-37757, 1:1000, ThermoFisher), and TOP2a (20233-1-AP, 1:1000, ThermoFisher).

### Immunoprecipitation

IPs between p53 and Med17 were carried out using nuclear extracts. Nuclear extracts were prepared^68,69^ and equal amounts of protein were used. Antibodies used for IP were: Med17 (PA5-40839, 3ug, ThermoFisher Scientific), PLK3 (4896S, 3 µg, Cell Signaling), and p53 (FL-393, 3 µg, Santa Cruz, Biotechnology).

### Chromatin immunoprecipitation

ChIP were performed as described^20^. Antibodies used for ChIP include: p53 Abs (DO1 and FL-393, sc-126 and sc-6243, Santa Cruz), Med17 (PA5-40839, ThermoFisher Scientific), PLK3 (4896S, Cell Signaling), and normal mouse and rabbit IgG (sc-2025 and sc-2027, Santa Cruz). ChIP-re-ChIP was performed as described^70^. Briefly, equal amounts of extracts were incubated with a primary antibody at 4 °C overnight. Protein A agarose beads were added and allowed to rock at 4 °C for 1 h before being washed once with RIPA, once with high salt buffer, once with LiCl buffer, and once with 1X TE. Protein complexes were then eluted from the protein A agarose beads by incubation at 37 °C for 30 min with 10 mM DTT in 1X TE. The next IP was then set up with the second antibody and incubated at 4 °C overnight. Protein A agarose beads were added and allowed to rock at 4 °C for 1 h before being washed once with RIPA, once with high salt buffer, twice with LiCl buffer, and twice with 1X TE. Regular ChIP procedure was followed afterwards. QPCR was used to quantify precipitated DNA using promoter specific primers (Supplementary Data 6).

### cDNA preparation and quantitative PCR

RNA was extracted and cDNA was prepared following published methods^71^. cDNA was synthesized using SuperScript III Reverse Transcriptase (ThermoFisher Scientific) and qPCR was used to measure transcript levels of GOF p53-activated genes using specific primers (Supplementary Data 6). Individual and pooled PLK3 siRNA was obtained from Horizon Discovery.

### RNA Sequencing

Total RNA was prepared using Trizol reagent (Invitrogen) from two clones. Total RNA was sent to the Donnelly Sequencing Centre (University of Toronto, Toronto, Ontario, Canada), who carried out the remaining procedures. mRNA libraries were generated from 1 µg of total RNA using Illumina TruSeq RNA Sample Prep V2 kits (RS-122-2001) according to the manufacturer’s directions. Sequencing was completed on the Illumina HiSeq2500 platform using Version 3 chemistry and reagents. Single-read data (50 bp) were processed with RTA 1.17.21.3, HCS 2.0.10.0 and aligned with CASAVA V1.8.2 secondary analysis package (Illumina, San Diego, CA). Fastq files were analyzed using DNAStar ArrayStar version 16.

### Immunohistochemistry

Staining was performed on formalin-fixed paraffin embedded lung tissue specimens as described^72^. Tissue embedding and sectioning was performed by the VCU Macromolecule Core laboratory. Immunohistochemistry was performed using a Vectastain ABC Elite kit (Vector Labs) following the manufacturer’s instructions. Sections were counterstained using Hematoxylin (Vector Labs) and mounted using Permount (Fisher). Images were viewed using a Labophot-2 microscope, captured using a 1080p HD 6MP microscope camera and processed using IS Capture software. Fluorescent stained sections were counterstained using DAPI (Sigma) and mounted using ProLong Gold Anti-fade (ThermoFisher). They were captured using a Zeiss LSM700 microscope and processed using Zeiss Zen software (2.3, 2016). Antibodies used were as follows: p53 (FL-393, 1:100, sc-6243, Santa Cruz Biotechnology) and TTF-1 (sc-53136, 1:100, Santa Cruz Biotechnology) were used for immunohistochemistry; SPC Antibody (FL-197): sc-13979, 1:100 and CCSP (CC10 Antibody (T-18): sc-9772, 1:100, Santa Cruz Biotechnology) were used for immunofluorescence.

### DNA replication origin firing

DNA replication origin firing was determined by DNA fiber spreading following published protocols^73^. In short, cells were sequentially pulse-labeled with nucleotide analogs IdU and CIdU, collected by trypsinization, and genomic DNA was aligned on slides by fiber spreading. The slides were dried and fixed in 3:1 methanol:acetic acid and dried overnight. DNA fibers on slides were acid treated (2.5 N HCl, 30 min) and blocked in 2% BSA in PBS, then stained with primary antibodies against IdU and CIdU. Slides were then stained with fluorescently labeled secondary and tertiary antibodies, washed, and mounted in antifade. Images were collected by confocal microscopy using a Zeiss LSM700 microscope. Approximately 200 fibers were scored and analyzed from each sample using ImageJ software (1.51k).

### Animal study approval

All animal studies were approved by the Institutional Animal Care and Use Committee at Virginia Commonwealth University. Mice were housed at the animal facility of Virginia Commonwealth University, Virginia, USA. Five-week-old female 129/Sv mice were used to generate the transgenic mice and 4-week-old female Nu/J mice were used in the studies. They were kept under clean conditions in 12/12 light/dark cycle, 65–75 °F and 40–60% humidity.

### Statistical analysis

All experiments had at least one additional independent repeat with similar results and all statistical analyses were calculated using an unpaired, two-tailed, equal variance Student’s *t*-test or a one-way ANOVA using GraphPad Prism 7.03. Standard error was calculated for all experiments and plotted as error bars. Data were considered significant if the *p*-value was below 0.05.

### Reporting summary

Further information on research design is available in the Nature Research Reporting Summary linked to this article.

## Supplementary information

Supplementary Information

Description of Additional Supplementary Files

Supplementary Data 1

Supplementary Data 2

Supplementary Data 3

Supplementary Data 4

Supplementary Data 5a

Supplementary Data 5b

Supplementary Data 6

Reporting Summary

## Data Availability

RNA-seq data are available in the public repository ArrayExpress under accession number E-MTAB-9833. All the other data supporting the findings of this study are available within the article, the Supplementary information file, and the Source data file. A Reporting summary for this article is available as Supplementary information file. Source data are provided with this paper.

## Source data

Source Data
