# Supplementary material for: The oncogenicity of tumor-derived mutant p53 is enhanced by the recruitment of PLK3
Source: Nat Commun. 2021 Jan 29;12:704. doi: 10.1038/s41467-021-20928-8 (PMC7846773; doi:10.1038/s41467-021-20928-8)
Supplement: Supplementary file 1 — Supplementary Information [file 41467_2021_20928_MOESM1_ESM.pdf]

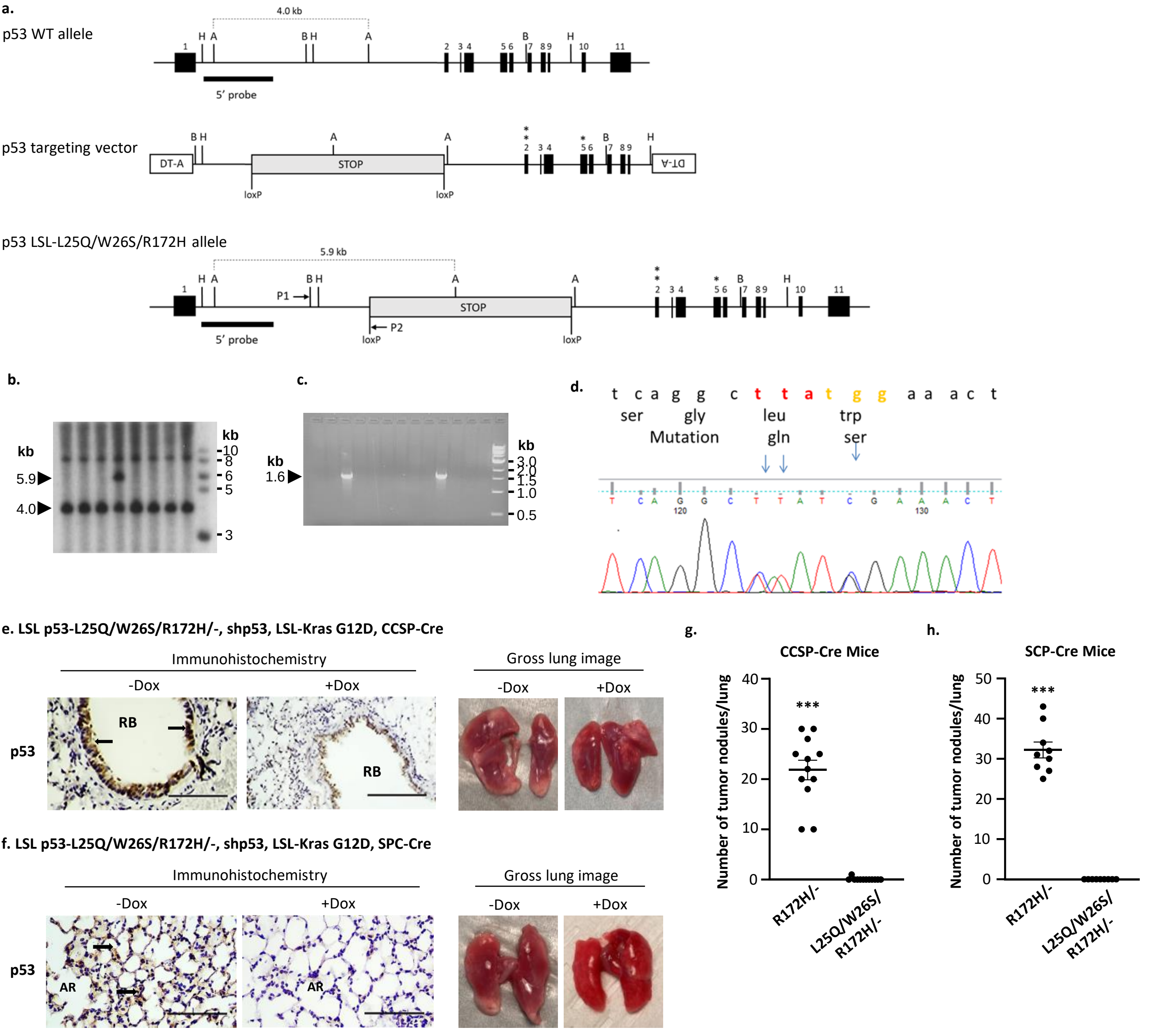

Supplementary Figure 1. Construction of Triple knock-in p53-L25Q/W26S/R172H mouse. A. Schematics of WT p53 allele, p53 targeting vector, and recombined allele are shown. The p53 LSL-L25Q/W26S/172H targeting vector contains 7.2 kb of genomic *p53* sequence (including exons 2-9), extending from the BamHI site in intron 1 to the HindIII site in intron 9. The vector also contains a Lox-Stop-Lox (LSL) cassette inserted into an XhoI site in intron 1 [13]. The LSL cassette contains a PGK/puromycin positive selection cassette, as well as an array of SV40 polyA regions that efficiently blocks expression of the allele until deleted by Cre recombinase. The targeting vector also contains two terminal MC1/DT-A (diphtheria toxin) negative selection cassettes. The L25Q/W26S mutations (in exon 2) and the R172H mutation (in exon 5) are indicated by asterisks. The positions of the PCR primers (P1 and P2) and the 5'-flanking Southern probe are indicated. Restriction sites are indicated: AflII (A), BamHI (B), and HindIII (H). Not all AflII sites are shown. **B.** Southern blot screening of AflII digests of ES clones utilized a 5'-flanking *p53* probe, with the WT p53 and p53 LSL-L25Q/W26S/172H alleles distinguished by bands of 4.0 and 5.9 kb, respectively. **C.** Long-range PCR screening of ES clones utilized a 5'-flanking *p53* primer (P1: 5'-AAGCCTCGAAGTAAGTTGGATGCC-3') in combination with an LSL primer (P2: 5'-TCTAATTCCATCAGAAGCTGGTCG-3'), with the p53 LSL-L25Q/W26S/172H allele identified by a PCR product of 1.6 kb. **D.** Sequencing of tail DNA showed the presence of mutations. The P53 L25Q/W26S/R172H/- mouse model was constructed in the presence of K-Ras as well, [LSL-P53 L25Q/W26S/R172H/-, LSL K-Ras (G12D), CCSP-Cre] by crossbreeding with LSL K-Ras (G12D). **E.** LSL p53 L25Q/W26S/R172H/-, shp53, K-Ras, CCSP-Cre mouse line. Representative images (Magnification: 20x, Scale bar: 100µM) of the CCSP mouse line showing lung Club cells immunostained for p53; +Dox shows a decrease in p53 expression indicated by reduced staining intensity; gross lung image indicates no tumor formation. Rb indicates respiratory bronchiole. **F.** LSL p53-L25Q/W26S/R172H/-, shp53, K-Ras, SPC-Cre mouse line. Representative images (Magnification: 20x, Scale bar: 100µM) of SPC mouse line showing lung Alveolar cells immunostained for p53 and gross lung image indicates no tumor formation. AR indicates alveolar region. **G.** Quantification of tumor nodules from lungs of LSL p53-R172H/-, shp53, K-Ras, CCSP-Cre and LSL p53 L25Q/W26S/R172H/-, shp53, K-Ras, CCSP-Cre mice (n=12 mice). Lungs were sectioned, stained with H&E, and every fifth slide was used to count tumor nodules. ***p<1E-10 **H.** Quantification of tumor nodules from lungs of LSL p53-R172H/-, shp53, K-Ras, SPC-Cre and LSL p53 L25Q/W26S/R172H/-, shp53, K-Ras, SPC-Cre mice (n=9 mice). Lungs were sectioned, stained with H&E, and every fifth slide was used to count tumor nodules. Data is presented as the mean ± SEM. ***p<1E-10

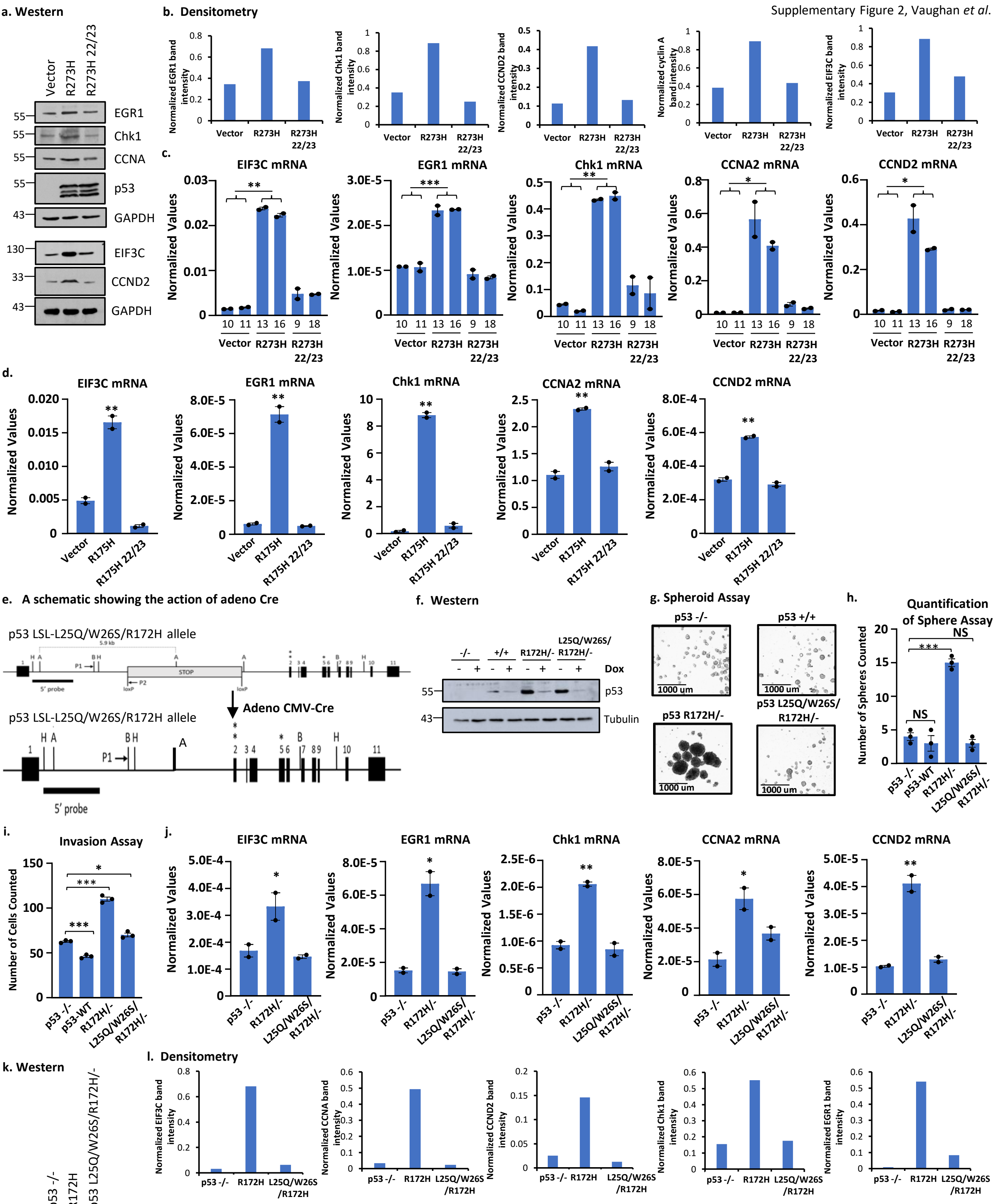

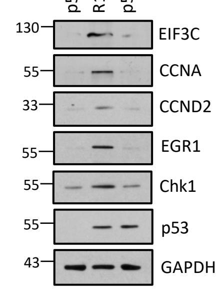

Supplementary Figure 2. Up-regulation of many GOF p53 inducible genes requires an intact GOF p53 transactivation domain. A. Western blot showing the level of p53 inducible proteins in H1299 cells expressing different p53 mutant derivatives. **B.** Densitometry calculated showing band intensities from the Western blots shown in A. **C.** QPCR verification of some genes from Supplementary Table 1 modulated by p53-R273H and -R273H L22Q/W23S. Data are normalized to GAPDH and is presented as the mean ± SEM (n=2 independent cell clones across 2 experiments). A two-sided Student's *t* test was performed and EIF3C **p=0.001, EGR1 ***p=8.4E-5, Chk1 **p=0.001, CCNA2 *p=0.02, CCND2 *p=0.03. **D.** QPCR verification of genes modulated by p53-R175H and -R175H L22Q/W23S. The selected genes are identical to those in C. Data are normalized to GAPDH and is presented as the mean ± SEM (n=3). A two-sided Student's *t* test was performed. **E.** A schematic showing the action of adeno Cre to explain generation of lung cells homogenously expressing mutant p53. LSL cassette is removed by Cre. **F.** Western blot showing the expression level of p53 in mouse cells with and without addition of Dox to induce shp53 already present within the cells. **G.** Spheroid growth assay for murine cells with genotypes p53 -/-, p53 +/+, p53 R172H/- and p53-L25Q/W26S/R172H/-. Best colonies are formed in p53-R172H cells. Colony formation ability seems to be compromised by TAD mutations. **H.** Quantification of spheroid growth assay for murine cells with genotypes p53 -/-, p53 +/+, p53 R172H/- and p53-L25Q/W26S/R172H/-. Data is presented as the mean ± SEM (n=3). A two-sided Student's *t* test was performed. **I.** Invasion assay performed on cells used to make spheroids in G. Data is presented as the mean ± SEM (n=3). A two-sided Student's *t* test was performed. **J.** Expression level of genes checked in p53 -/-, p53 R172H/-, and p53-L25Q/W26S/R172H/- mouse cells by QPCR of cDNA prepared from RNA from the mouse cells shown G. Data are normalized to GAPDH and is presented as the mean ± SEM (n=3). A two-sided Student's *t* test was performed. **K.** Western blot showing the level of p53 inducible proteins in murine cells with genotypes p53 -/-, p53 172H/- and P53 L25Q/W26S/R172H/-/-. **L.** Densitometry calculated showing band intensities from the Western blots shown in K. *p-value < 0.05, **p-value < 0.01, and ***p-value < 0.001. NS-no statistically significant difference from control.

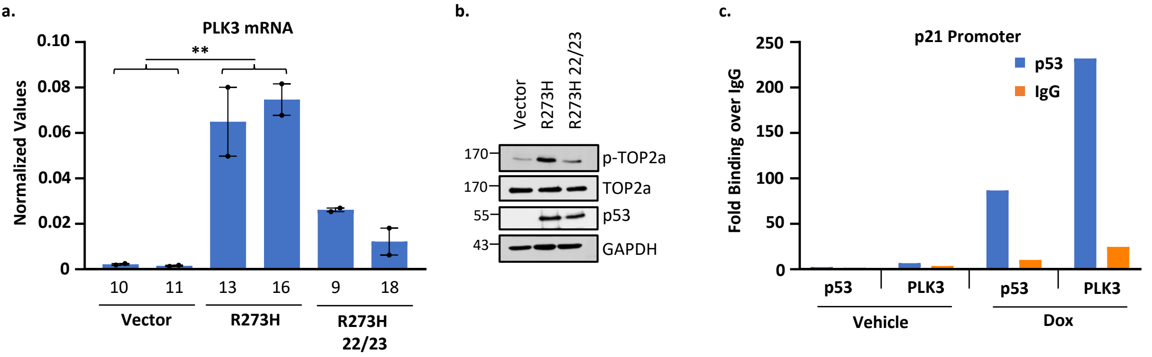

Supplementary Figure 3. PLK3 downregulation affects substrate expression. A. PLK3 mRNA was measured in H1299 cells transfected with empty vector or stably expressing p53-R273H and -R273H L22Q/W23S. Data is presented as the mean ± SEM (n=2 independent cell clones across 2 experiments). A two-sided Student's *t* test was performed and **p=0.005. **B.** Western blot analysis showing a PLK3 substrate, TOP2a, in H1299 cells transfected with empty vector or stably expressing p53-R273H and -R273H L22Q/W23S. **C.** ChIP-reChIP of H1299 cells stably transfected with empty vector or expressing p53-R273H after doxorubicin treatment is shown to look at the p53-PLK3 interaction on a mutant p53 inducible gene promoter after stress conditions.

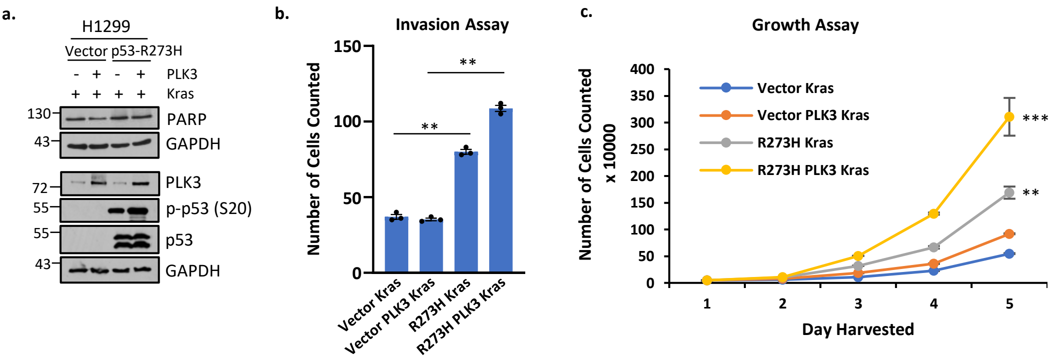

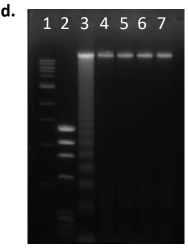

Supplementary Figure 4. PLK3 overexpression positively affects growth properties of the cell. A. Western blot analysis of H1299 cells stably transfected with vector control or expressing p53-R273H along with PLK3 and or Kras G12D. **B.** Invasion assay of cells shown in A. Data is presented as the mean ± SEM (n=3). A two-sided Student's *t* test was performed. **C.** Growth assay of cells shown in A. Data is presented as the mean ± SEM (n=3). A two-sided Student's *t* test was performed and ***p-value < 6.9E-5. **D.** DNA ladder assay on cells shown in A-C. Loading order is: 1-1kb DNA ladder, 2-PhiX HaeIII DNA ladder, 3-U937 apoptotic cell DNA (positive control), 4-H1299 pCMV Bam neo + pSport6 Kras G12D, 5-H1299 pCMV Bam neo + Flag PLK3 + pSport6 Kras G12D, 6- H1299 pCMV Bam neo p53-R273H + pSport6 Kras G12D, and 7-H1299 pCMV Bam neo p53-R273H + Flag PLK3 + pSport6 Kras G12D.
