# Supplementary material for: The oncogenicity of tumor-derived mutant p53 is enhanced by the recruitment of PLK3
Source: Nat Commun. 2021 Jan 29;12:704. doi: 10.1038/s41467-021-20928-8 (PMC7846773; doi:10.1038/s41467-021-20928-8)
Supplement: Supplementary file 2 — Description of Additional Supplementary Files [file 41467_2021_20928_MOESM2_ESM.pdf]

Description of Additional Supplementary Files

File Name: Supplementary Data 1
Description: Abbreviated list of genes that are activated by p53-R273H and require an intact transactivation domain.

File Name: Supplementary Data 2
Description: Complete list of genes that are activated by p53-R273H and require an intact transactivation domain.

File Name: Supplementary Data 3
Description: Complete list of genes that have p53 bound to the promoter. Complete list of genes that are activated by p53-R273H and do not require an intact transactivation domain.

File Name: Supplementary Data 4
Description: DAVID analysis of Supplementary Tables 1-3.

File Name: Supplementary Data 5a
Description: RNA seq analysis from mouse lung RNA isolated mouse cells made near homogenously expressing p53 -/-, p53-R172H/- and p53-L25Q/W26S/R172H/-.

File Name: Supplementary Data 5b
Description: DAVID analysis of the RNA seq data presented in Suppplementary Table 5a.

File Name: Supplementary Data 6
Description: List of primers used for qPCR.
