# Supplementary material for: The oncogenicity of tumor-derived mutant p53 is enhanced by the recruitment of PLK3
Source: Nat Commun. 2021 Jan 29;12:704. doi: 10.1038/s41467-021-20928-8 (PMC7846773; doi:10.1038/s41467-021-20928-8)
Supplement: Supplementary file 10 — Reporting Summary [file 41467_2021_20928_MOESM10_ESM.pdf]

nature research

Corresponding author(s): Sumitra Deb

Last updated by author(s): Dec 14, 2020

Reporting Summary

Nature Research wishes to improve the reproducibility of the work that we publish. This form provides structure for consistency and transparency in reporting. For further information on Nature Research policies, see our Editorial Policies and the Editorial Policy Checklist.

Statistics

For all statistical analyses, confirm that the following items are present in the figure legend, table legend, main text, or Methods section.

n/a	Confirmed	
☐	☒	The exact sample size (*n*) for each experimental group/condition, given as a discrete number and unit of measurement
☐	☒	A statement on whether measurements were taken from distinct samples or whether the same sample was measured repeatedly
☐	☒	The statistical test(s) used AND whether they are one- or two-sided<br>*Only common tests should be described solely by name; describe more complex techniques in the Methods section.*
☐	☒	A description of all covariates tested
☒	☐	A description of any assumptions or corrections, such as tests of normality and adjustment for multiple comparisons
☐	☒	A full description of the statistical parameters including central tendency (e.g. means) or other basic estimates (e.g. regression coefficient) AND variation (e.g. standard deviation) or associated estimates of uncertainty (e.g. confidence intervals)
☐	☒	For null hypothesis testing, the test statistic (e.g. *F*, *t*, *r*) with confidence intervals, effect sizes, degrees of freedom and *P* value noted<br>*Give P values as exact values whenever suitable.*
☒	☐	For Bayesian analysis, information on the choice of priors and Markov chain Monte Carlo settings
☒	☐	For hierarchical and complex designs, identification of the appropriate level for tests and full reporting of outcomes
☒	☐	Estimates of effect sizes (e.g. Cohen's *d*, Pearson's *r*), indicating how they were calculated

Our web collection on statistics for biologists contains articles on many of the points above.

Software and code

Policy information about availability of computer code

Data collection	Zeiss Zen software (2.3, 2016) was used to acquire fluorescent images, IS Capture software (Version 4.1.3) was used to acquire IHC stained tissue sections, RNA sequencing was completed on the Illumina HiSeq2500 platform
Data analysis	ImageJ software (1.51k) was used to score and analyze DNA fibers and DNAStar ArrayStar 14 was used to analyze RNA-sequencing data. GraphPad Prism 7.03 was used for statistical analysis.

For manuscripts utilizing custom algorithms or software that are central to the research but not yet described in published literature, software must be made available to editors and reviewers. We strongly encourage code deposition in a community repository (e.g. GitHub). See the Nature Research guidelines for submitting code & software for further information.

Data

Policy information about availability of data

All manuscripts must include a data availability statement. This statement should provide the following information, where applicable:

- Accession codes, unique identifiers, or web links for publicly available datasets
- A list of figures that have associated raw data
- A description of any restrictions on data availability

The source data underlying Figs. 2b-e; 3a-d; 4a-h; 5a, c-h; 6a-e; 7a-d, f-g; 8a-f and Supplementary Figs. 1b, c; 2a-d, f, h-l; 3a-c; 4a-d, are provided as Source Data file. RNA-seq data is available in the public repository ArrayExpress under accession number E-MTAB-9833 [https://www.ebi.ac.uk/arrayexpress/experiments/E-MTAB-9833/]. All the other data supporting the findings of this study are available within the article, the Supplementary Information file, and the Source Data file. A Reporting Summary for this article is available as Supplementary Information file.

Field-specific reporting

Please select the one below that is the best fit for your research. If you are not sure, read the appropriate sections before making your selection.

- [x] Life sciences
- [ ] Behavioural & social sciences
- [ ] Ecological, evolutionary & environmental sciences

For a reference copy of the document with all sections, see nature.com/documents/nr-reporting-summary-flat.pdf

Life sciences study design

All studies must disclose on these points even when the disclosure is negative.

Sample size	No statistical methods were used to predetermine sample size. Sample sizes were chosen based on prior knowledge in the respective experiments and their intrinsic variability as performed in previous studies. Experiments were performed in triplicate and biological significance was only ascribed when the observed effects met a p-value < 0.05.
Data exclusions	No data were excluded from analyses.
Replication	All experiments analyzed by immunoblot, immunofluorescence, immunohistochemistry, and immunoprecipitation were performed with at least three replicates and representative results are shown. All findings were successfully reproduced in independent experiments.
Randomization	Samples were not randomized as no experimental groups were used in this study. In each experiment, we had cell lines (or mice) that did not contain mutant p53 and that was used to compare against cells (or mice) that had mutant p53. In the case of H1975 PLK3 knockout cells, we used a H1975 control cell line to compare against.
Blinding	Investigators were not blinded during the completion of this study because collection and analysis of the presented data was not prone to any bias. Furthermore, the readout of experiments was precise and based on quantitative measurements and not based on subjective assessments.

Reporting for specific materials, systems and methods

We require information from authors about some types of materials, experimental systems and methods used in many studies. Here, indicate whether each material, system or method listed is relevant to your study. If you are not sure if a list item applies to your research, read the appropriate section before selecting a response.

Materials & experimental systems

n/a	Involved in the study	
[ ]	[x]	Antibodies
[ ]	[x]	Eukaryotic cell lines
[x]	[ ]	Palaeontology and archaeology
[ ]	[x]	Animals and other organisms
[x]	[ ]	Human research participants
[x]	[ ]	Clinical data
[x]	[ ]	Dual use research of concern

Methods

n/a	Involved in the study	
[x]	[ ]	ChIP-seq
[x]	[ ]	Flow cytometry
[x]	[ ]	MRI-based neuroimaging

Antibodies

Antibodies used	For IHC/IF:<br>p53 (FL-393; sc-6243, Santa Cruz Biotechnology)<br>TTF-1 (sc-53136, Santa Cruz Biotechnology)<br>SPC Antibody (FL-197; sc-13979, Santa Cruz Biotechnology)<br>IdU (Anti-BrdU, B44, 347580, BDBiosciences)<br>CldU (Anti-BrdU, 347580, OBT0030G, BioRad)<br>CCSP (CC10 Antibody (T-18); sc-9772, Santa Cruz Biotechnology)<br>For IP:<br>Med17 (PA5-40839, Thermo Fisher Scientific)<br>PLK3 (4896S, Cell Signaling)<br>p53 (PAb 1801, Santa Cruz, Biotechnology)<br>Normal mouse IgG (sc-2025, Santa Cruz, lot G2020)<br>Normal rabbit IgG (2729, Cell Signaling, lot 27)<br>For IB:<br>p53 (PAb 1801), purified from hybridoma cells<br>EIF3C (2068S, Cell Signaling)<br>EGR1 (4154S, Cell Signaling)

cyclin A (sc-239, Santa Cruz)
cyclin D2 (2924S, Cell Signaling)
GAPDH (sc-32233, Santa Cruz)
Tubulin (2148S, Cell Signaling)
p-p53 Ser6 (9285S, Cell Signaling)
p-p53 Ser9 (9288S, Cell Signaling)
p-p53 Ser15 (9286S, Cell Signaling)
p-p53 Ser20 (9287S, Cell Signaling)
PLK3 (4896S, Cell Signaling)
Chk1 (2360S, Cell Signaling)
Flag (F1804, Sigma Aldrich)
PARP (9532, Cell Signaling)
p-TOP2a (PA5-37757, ThermoFisher)
TOP2a (20233-1-AP, ThermoFisher)

Validation

All commercial antibodies used have been validated by the vendors and validation data are available on the manufacturer's website.
For IHC/IF:
p53: Validated by manufacturer using paraffin-embedded human breast carcinoma tissue.
TTF-1: Validated by manufacturer using immunoperoxidase staining of formalin fixed, paraffin-embedded human thyroid tissue.
SPC Antibody: Validated by manufacturer using immunoperoxidase staining of formalin fixed, paraffin-embedded human lung tissue.
CCSP: Validated by manufacturer using immunoperoxidase staining of formalin fixed, paraffin-embedded human bronchus tissue.
IDU: Supplier webpage. Validated in immunofluorescence. https://www.bdbiosciences.com/ds/is/tds/23-1349.pdf
CldU: Supplier webpage. Validate in immunofluorescence. https://www.bio-rad-antibodies.com/static/datasheets/obt00/brdu-antibody-bu1-75-icr1-obt0030g.pdf
For IP:
Med17: Validated by manufacturer using human HepG2 cell lysate
PLK3: Validated by manufacturer extracts from 293T cells transfected with a human PLK3 construct
p53: Validated by manufacturer using human HepG2 cell lysate
For IB:
p53 (pAb 1801) : Validated by group in our previous work (PMID: 26820293, 23264849, 22198284).
EIF3C: Validated by manufacturer using MCF-7, A204, RD, K562 cell lysates.
EGR1: Validated by manufacturer using serum starved, NGF-treated PC-12 cell lysates.
Cyclin A: Validated by manufacturer using K-562 whole cell lysates.
Cyclin D2: Validated by manufacturer and various publications.
GAPDH: Validated by manufacturer using Hep G2 and A549 cell lysates.
Tubulin: Validated by manufacturer using HeLa, NIH/3T3, C6 and COS-7 cell lysates.
p-p53 Ser6: Validated by manufacturer using COS cells treated with UV or MMS.
p-p53 Ser9: Validated by manufacturer using COS cells treated with UV or MMS.
p-p53 Ser15: Validated by manufacturer using HT29 cells treated with UV.
p-p53 Ser20: Validated by manufacturer using COS cells treated with UV or MMS.
PLK3: Validated by manufacturer using extracts from 293T cells transfected with a human PLK3 construct.
Chk1: Validated by manufacturer using extracts from A431, NIH/3T3, HeLa cell lysates.
Flag: Validated by manufacturer using CHO lysates to identify spiked Flag protein.
PARP: Validated by manufacturer using extracts from THP-1 cells, untreated or treated with TNF-α and cycloheximide as well as control extracts from SW620 and A20 cell lines.
p-TOP2a: Validated by manufacturer using extracts from HepG2 cells treated with Ca^{2+}
TOP2a: Validated by manufacturer using extracts from HeLa cells.

Eukaryotic cell lines

Policy information about cell lines

Cell line source(s)

H1299 (human, ATCC CRL-5803)
H1975 (human, ATCC CRL-5908)

Authentication

None of the cell lines have been further authenticated.

Mycoplasma contamination

Cell lines were not tested for mycoplasma contamination but no indication of contamination was observed.

Commonly misidentified lines
(See ICLAC register)

No commonly misidentified cell lines were used.

Animals and other organisms

Policy information about studies involving animals; ARRIVE guidelines recommended for reporting animal research

Laboratory animals

4-week old female Nonobese diabetic/severe combined immunodeficiency (NOD/SCID) and athymic nude (Nu/J) mice were used in this study for xenograft tumor development. 5-week old female 129/Sv mice were used to generate the transgenic mice used in the studies. Mice were housed at the animal facility of Virginia Commonwealth University, Virginia, USA. They were kept under clean conditions in 12/12 light/dark cycle, 65-75°F and 40-60% humidity.

Wild animals	No wild animals were used in this study.
Field-collected samples	No field-collected samples were used in this study.
Ethics oversight	The mouse work was performed under the study protocol AM10234, as approved by Virginia Commonwealth University's Institutional Animal Care and Use Committee.

Note that full information on the approval of the study protocol must also be provided in the manuscript.
